# Quantifying Protein Homodimer Affinities and the Effect of Molecular Glues and Interface Residues Using Native Mass Spectrometry

**DOI:** 10.1021/jacs.5c18602

**Published:** 2026-04-01

**Authors:** Jonathan Schulte, Eric Schwegler, Ute A. Hellmich, Nina Morgner

**Affiliations:** † Institute of Physical and Theoretical Chemistry Goethe-University, Max-von-Laue-Str. 9, Frankfurt 60438, Germany; ‡ Faculty of Chemistry and Earth Sciences Institute of Organic Chemistry and Macromolecular Chemistry, Friedrich-Schiller-University, Jena 07743, Germany; § Center for Biomolecular Magnetic Resonance (BMRZ) Goethe-University, Frankfurt 60438, Germany; ∥ Cluster of Excellence Balance of the Microverse Friedrich-Schiller-University, Jena 07743, Germany; ⊥ Cluster of Excellence SubCellular Architecture of Life (SCALE), Goethe-University, Frankfurt 60438, Germany

## Abstract

Biological processes rely on finely tuned homo- and heteromeric interactions between (biomacro)­molecules. The strength of an interaction, typically given by the dissociation constant (*K*_D_), plays a crucial role in basic research and must be monitored throughout the development of drugs and agrochemicals. An ideal method for *K*_D_ determination is applicable to various analytes with a large range of affinities, tolerates complex matrix compositions, does not require labeling, and simultaneously provides information on the structural integrity of the binding partners. Native mass spectrometry meets these criteria but typically struggles with homooligomeric complexes due to overlapping mass signals. To overcome this, we resolve monomer/dimer contributions to overlapping MS-peaks by separately analyzing the charge state distribution of each oligomeric species via sample dilution and covalent cross-linking. Following this approach, we show that quantitative laser-induced liquid bead ion desorption mass spectrometry (qLILBID-MS) accurately captures the affinities of Bovine Serum Albumin (BSA) and chemically induced dimers of Tryparedoxin (Tpx), an oxidoreductase from human pathogenic *Trypanosoma brucei* parasites, with various molecular glues and homodimer affinities. Conveniently, qLILBID-MS requires a fraction of sample used by other methods such as isothermal titration calorimetry (ITC) and yields previously inaccessible protein homodimer *K*_D_s in the high micromolar range, which allowed us to monitor the gradual decrease in homodimer affinity via mutation of crucial dimer interface contacts. Overall, qLILBID-MS is a sensitive, robust, fast, scalable, and cost-effective alternative to quantify protein/protein interactions, that can accelerate contemporary drug discovery workflows, e.g. the efficient screening for proximity inducing molecules like proteolysis targeting chimera (PROTACs) and molecular glues.

## Graphical abstract

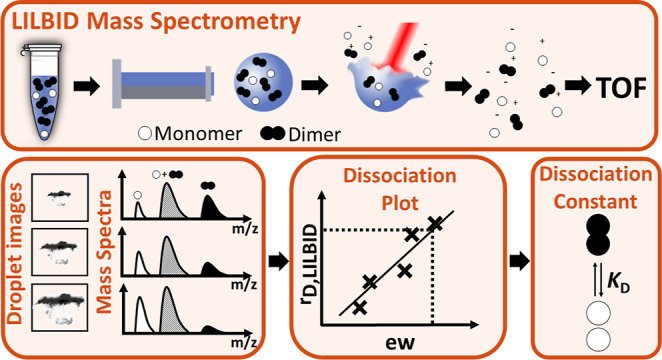

## Introduction

All biological processes including enzymatic catalysis, signal transduction, cellular localization, or the assembly of functional molecular machines, are driven by interactions between (biomacro)­molecules across a wide range of affinities.^1–3^ The deliberate induction of protein/protein interactions (PPIs) via external stimuli such as light or addition of small molecules is a versatile strategy to exert spatiotemporal control over biological processes.^4,5^ Chemically induced dimerization (CID) of proteins has emerged as a particularly powerful strategy to address therapeutic targets in inflammatory, neurodegenerative and infectious diseases that were previously deemed “undruggable”.^6,7^ Prominent examples are so-called molecular glue degraders and proteolysis-targeting chimeras (PROTACs), which initiate the degradation of a biological target via induced proximity to an E3 ubiquitin ligase.^8^ The identification of such proximity inducing molecules, and in general the description of biomacromolecular interactions, requires the ability to precisely and efficiently quantify binding affinities, typically through the determination of the corresponding dissociation constants (*K*_D_). This is crucial to elucidate structure activity relationships (SARs), to identify and understand key molecular interactions and to provide the basis for drug development.^1,9,10^

Established techniques for protein affinity determination, such as surface plasmon resonance (SPR) or isothermal titration calorimetry (ITC), offer valuable insights but typically involve substantial analyte quantities or elaborate sample preparation.^11–14^ This might include an additional step to elucidate biomolecular complex stoichiometries to obtain reliable *K*_D_ values.^1,15,16^

Fluorescence-based methods are versatile and require significantly less material but are dependent on the introduction of fluorophores through non-native tags and/or specific labeling approaches, often enabled by mutagenesis. Finally, tag-free methods such as NMR spectroscopy are less invasive and offer direct insights into analyte integrity but again require isotope labeling and comparatively high amounts of sample. In all cases, homooligomeric systems present particular challenges when aiming to quantify binding affinities, e.g. if the separation of binding partners is required prior to a measurement, as it is usually the case in titration-based methods.

Native mass spectrometry (MS) has become a powerful technique for simultaneously assessing the composition, stoichiometry, and binding affinity of macromolecular complexes without the need for labeling, large sample amounts or extensive sample preparation.^17,18^ Its gentle ionization process preserves noncovalent interactions, allowing intact complexes to be detected directly.^17,19^ For instance, dissociation constants (*K*_D_) can be determined, using nanoelectrospray ionization (ESI)-MS titration, where mass spectra are recorded at different analyte concentrations.^20,21^ Competitive ligands with known affinities may be added to refine specificity and validate binding interactions.^22,23^ An example for a competitive ligand method is the catch-and-release (CaR) ESI-MS approach developed by the Klassen group.^24,25^ It enables competitive screening of ligand libraries by monitoring which binders remain associated with a protein target during controlled gas-phase dissociation.^25,26^ It is particularly useful for identifying moderate-to-strong binders from complex mixtures with low sample consumption and high throughput. However, it typically yields relative affinities and requires ESI-MS-compatible, volatile buffers.^24–26^ The intact transition epitope mapping (ITEM) concept by the Glocker group combines native MS with top–down fragmentation to map protein–ligand interaction sites and deduce binding characteristics.^27–29^ ITEM is especially powerful for identifying epitopes and interaction interfaces at high structural resolution and low material input.^27,28,30^ With the expansion to ITEM-TWO (thermodynamic weak-force order) the Glocker group enabled the method to determine gas phase *K*_D_s, which scaled comparable to solution *K*_D_s.^31^ Yet, ITEM also relies on volatile buffers which can affect the thermodynamic equilibrium and hence the dissociation constant.^27–31^

There have been a multitude of other MS based methods to determine affinities or screen ligands, such as TNT-MS introduced by the Lermyte group,^32^ SLOMO by the Klassen group^33^ or the GAP sampler by the Zenobi group^34^ and many others.^35^

These native MS-based methods usually offer various advantages compared to conventional methods,^36,37^ including high sensitivity, minimal sample consumption, concurrent determination of identity and integrity of an analyte, simultaneous determination of complex stoichiometry, and the ability to study biomolecular interactions in complex (biological) matrices.^15,35,38,39^ While these methods often allow for screening or the determination of additional structural information, they often require titration, competitive displacement and hence known binders, or labeling and are mostly restricted to volatile buffers as well as heterodimeric systems.

As an alternative technique we recently introduced quantitative laser-induced liquid bead ion desorption (qLILBID)-MS as a novel, native MS-based method for the analysis of stoichiometries and binding affinities of heterodimeric biomacromolecular complexes.^35,40^ This top–down method enables direct determination of the *K*_D_ value from a single sample, eliminating the need for separation of the binding partners and repeated measurements across varying analyte concentrations.^35,40^ Further, qLILBID tolerates a broad range of aqueous buffers, including nonvolatile and physiological compositions, and operates label-free with minimal sample consumption. Here, we extended this approach to homodimeric protein complexes, with *K*_D_ values in the low nanomolar to high micromolar range.

To establish qLILBID as a versatile tool to investigate diverse biomolecular complexes across a wide range of biologically relevant affinities, several challenges had to be overcome: In LILBID-MS, aqueous sample droplets are generated and transferred into vacuum. Each droplet is irradiated by an infrared (IR) laser pulse, which excites the OH vibration of water, resulting in explosive droplet expansion during which biomolecular complexes are desorbed and separated from some of their counterions. The mass to charge ratios of the ions generated in that way are then determined in a Time-of-Flight (ToF) analyzer (Figure 1A). While most laser energy is absorbed by the sample droplet liquid matrix, some energy induces dissociation of the non-covalent analyte complexes, an effect that becomes more pronounced at higher laser energies.^40,41^ This is the effect on which the qLILBID *K*_D_ determination is based. The challenge for reproducible measurements is that the energy uptake, and thus the degree of laser-induced complex dissociation (*r*_D,LILBID_), varies from droplet to droplet, due to unavoidable variations in sample droplet trajectories and velocities, thereby affecting the overall laser beam exposure of each droplet. To address this, we correlated the laser energy absorption with the respective droplet plume expansion for each droplet, quantified as the “explosion width” (ew) at 5 μs post-irradiation (Figure 1B).^29^ Together, the qLILBID mass spectra and the droplet explosion widths (ew) then enabled to directly correlate energy transfer and the degree of biomolecular complex dissociation (*r*_D,LILBID_), the basis for *K*_D_ determination (Figure 1C). Taking advantage of the linear relationship between energy uptake (∝ ew) and complex dissociation (*r*_D,LILBID_), the laser energy is ramped to determine *r*_D,LILBID_ for a range of droplet explosion widths (Figure 1D), allowing to deduce *K*_D_ values by comparing the analyte’s *r*_D,LILBID_ to a calibration plot, generated from *r*_D,LILBID_ values of calibration standards recorded under the same experimental conditions (Figure 1E).

**Figure 1:**
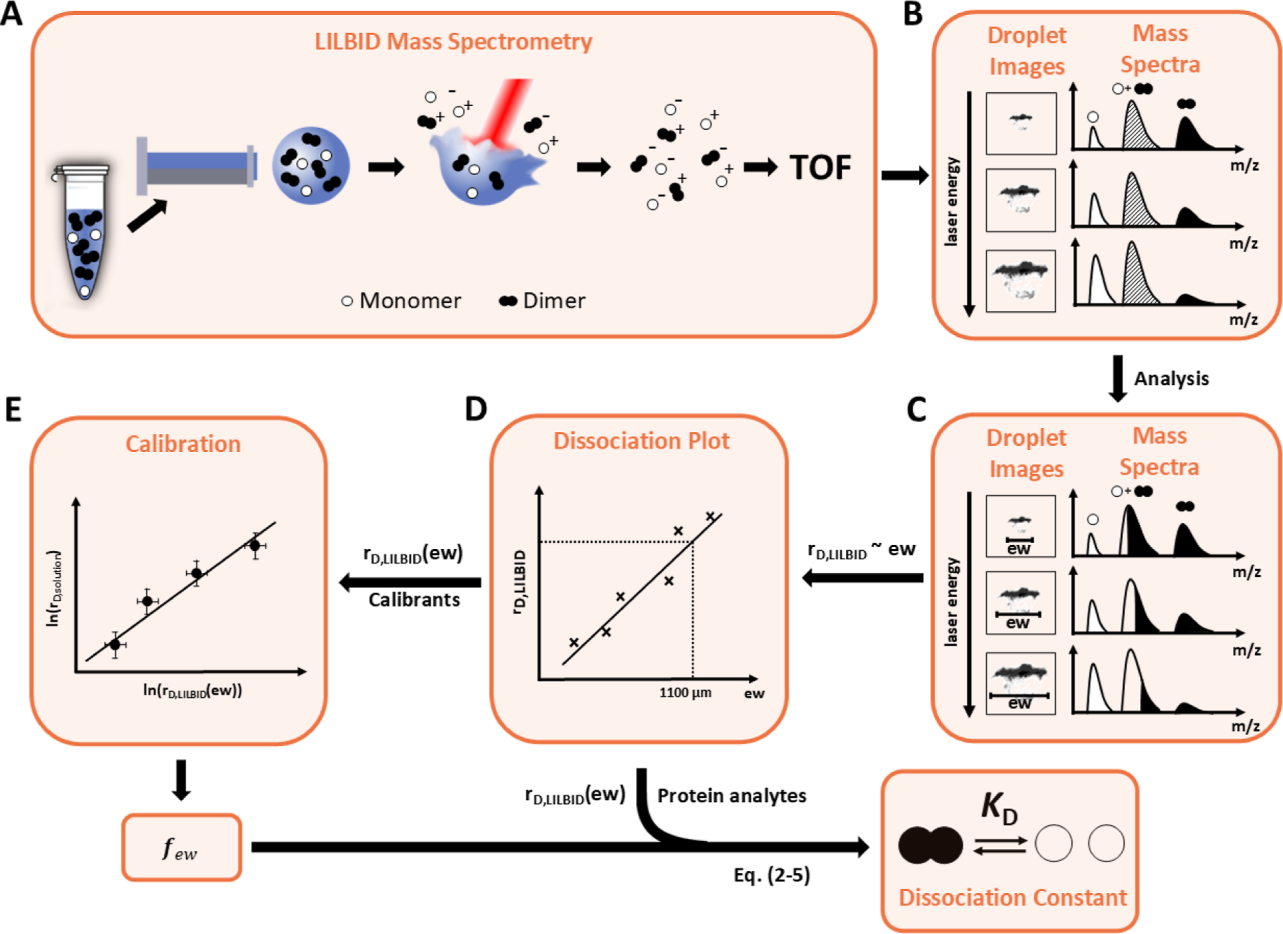
Workflow for quantitative LILBID-MS-based determination of dissociation constants (*K*_D_) of homodimeric biomacromolecules. (A, B) First, LILBID mass spectra and corresponding droplet explosion images are recorded at various droplet positions and laser energies. (C) Then, the ratio of dissociation (*r*_D,LILBID_) is calculated from the MS-peak integrals as the ratio of monomer signal to total signal (see eq 5). Deconvolution of overlapping charge states (e.g., singly charged monomer and doubly charged dimer) is achieved via dilution or cross-linking strategies (detailed in the main text), and droplet explosion widths (ew) are extracted via image analysis. (D) Plotting *r*_D,LILBID_ against explosion width yields a dissociation plot which is used to deduce *r*_D,LILBID_ for a certain ew (here: 1100 μm, details in main text) via linear regression. (E) For calibrants with known *K*_D_s, this value is used to generate a calibration plot correlating ln­(*r*_D,solution_) with ln­(*r*_D,LILBID_(ew)) which yields the calibration factor *f*_ew_ (see eq 6). This factor is then used to calculate *r*_D,solution_, and ultimately the dissociation constant *K*_D_, from *r*_D,LILBID_(ew) of analyte dimers with unknown affinities. Black, white, and hatched peaks in the spectra represent dimers, monomers, and mixed monomer/dimer peaks with overlapping charge states, respectively.

Although we previously showed that qLILBID-MS is applicable to determine the affinities of heterodimeric oligonucleotides (e.g., double stranded DNA) or RNA/protein complexes,^35,40^ homodimeric proteins present specific challenges. Here, the low charge state distributions generated in the LILBID process lead to peak overlap in the mass spectra between monomeric ions (M) and dimers (D) with double charge states (same mass/charge ratio (*m*/*z*) for M^*x*–^ and D^2*x*–^ ions), severely complicating monomer/dimer quantification. With two complementary approaches, i.e. complex dilution and covalent cross-linking using bovine serum albumin (BSA) as a benchmark, we show that MS peak overlap can be resolved.

To then put our approach to the test across a wide affinity range, we determined the *K*_D_ values of chemically induced homodimers of Tryparedoxin (Tpx), an essential oxidoreductase from human pathogenic *Trypanosoma brucei* (*T. brucei*) parasites.^42^ Covalent attachment of different molecular glues to the nucleophilic active site residue cysteine40 induces homodimerization of the protein with low to high micromolar affinities.^43,44^ Likewise, mutations in the Tpx dimer interface tune dimer affinity upon binding of the molecular glues.^45,46^ This system thereby allowed us to investigate the role of key interactions in the dimer interface using qLILBID.

Here, we establish qLILBID-MS as an accurate tool to capture *K*_D_ values of noncovalent protein homodimers with chemically diverse dimer interfaces, allowing us to sensitively resolve even small variations in *K*_D_ values using a comparatively small amount of unlabeled analyte, a key requirement for the reliable determination of biomacromolecular interactions in a physiological affinity range.

## Results and Discussion

### Correlating Ion Signal Ratios with Solution Ratios

The stability of a bimolecular complex in solution is typically given as dissociation constant *K*_D_, defined as

1 $$K_{D}=\frac{\left[M_{1}\right]\cdot \left[M_{2}\right]}{\left[D\right]}$$

with the concentrations of monomers (*M*_1_, *M*_2_) and dimers (*D*) present in a steady-state dissociation equilibrium

1a $$D⇌M_{1}+M_{2}$$

For homodimers, the definition of the dissociation constant (*K*_D_) changes to

2 $$K_{D}=\frac{{\left[M\right]}^{2}}{\left[D\right]}$$

as dimer formation solely depends on the concentration of the monomer (M)

2a D⇌2M

Due to the dissociation of the dimer into two identical monomers instead of two different compounds, the concentration ratio of monomer to dimer differs for homodimers and heterodimers at a given dissociation constant and analyte concentration (*c*_total_). To nonetheless establish a consistent workflow for homo- and heterodimers alike, we defined their ratio of dissociation in solution (*r*_D,solution_) as

3 $$r_{D,solution}=\frac{n\left(M_{1}\right)}{n\left(M_{1}\right)+n\left(D\right)}$$

with *n* as the number of monomers (*M*_1_) and dimers (*D*), respectively. Together with the total concentration of protein, either free or bound (*c*_total_), the *r*_D,solution_ can be used to calculate *K*_D_ values of heterodimers using eq 4a

4a $$K_{D,heterodimer}=\frac{r_{D,solution}^{2}\cdot c_{0}}{1-r_{D,solution}}$$

and *K*_D_ values of homodimers using eq 4b (for more details, see eqs S10–S16)­

4b $$K_{D,homodimer}=\frac{r_{D,solution}^{2}\cdot c_{0}}{r_{D,solution}^{2}-3r_{D,solution}+2}$$

As we have previously shown for dsDNA and protein/RNA heterodimers,^35^ LILBID-MS can accurately capture the thermodynamic equilibrium state in solution. It has to be noted that the ratio of monomeric versus overall signal in the LILBID spectra does not per se represent the solution ratio (and therewith *r*_D,solution_), since laser dissociation can increase the number of monomers appearing in the mass spectra. Nevertheless, *r*_D,solution_ can be correlated with the respective ratio in LILBID spectra (*r*_D,LILBID_), defined as

5 $$r_{D,LILBID}=\frac{\int M_{1}peaks}{\int M_{1}peaks+\int Dpeaks}$$

assuming the ionization efficiency of the different species is comparable. Hereby, “∫*X* peaks” is the peak integral of species *X*, here either *M*_1_ or *D*.

For the calculation of peak integrals, the ToF spectrum rather than the *m*/*z* spectrum was analyzed, since the ToF spectra reflect the temporal distribution of ion arrivals, effectively counting ions as a function of flight time, while the standardly used *m*/*z* spectra replace the ToF axis with *m*/*z* with (*m*/*z*)^2^ ∝ ToF. Therefore, the resulting peak integrals of the ToF spectra are better suited to quantify the corresponding species. Further details on the calculation of peak areas for oligonucleotides can be found in Young et al.^40^ and Schulte et al.^35^

To deduce the ratio of monomers in solution (*r*_D,solution_) from the qLILBID spectra, a correction factor had to be defined which correlates *r*_D,solution_ (taking only solution monomers into account) with *r*_D,LILBID_ (including solution monomers and monomers which are laser dissociation products) which is directly calculated from the LILBID spectra. To this end, the extent to which a given laser energy transfer level increases the signal stemming from monomeric species had to be determined via calibration. Here, we used a calibration set of dsDNAs with known *K*_D_ values (see below) and determined the calibration factor (*f*_ew_) for the given energy transfer.

Thus, based on *r*_D,LILBID_ at a given explosion width (ew), *r*_D,solution_ (eq 3) can be calculated using eq 6

6 $$\ln\left(r_{D,solution}\right)=\ln\left(r_{D,LILBID}\right)\cdot f_{ew}$$

with the corresponding calibration factor *f*_ew_.

In this study we used an explosion width of 1100 μm as reference.

6a $$\ln\left(r_{D,solution}\right)=\ln\left(r_{D,\mathrm{LILBID}}(1100\mu m)\right)\cdot f_{1100}$$

This allowed us to consider dissociation during the LILBID process, and determine *r*_D,solution_ and thus the *K*_D_ of a hetero- or homo-dimeric analyte using eqs 4a or 4b.

### Calibration

For calibration, we used a set of previously reported^40^ dsDNA heterodimers with nM to low μM affinities containing three different DNA strands with a length of 35 nucleobases (strA, strB, strC) and 9 complementary, shorter strands with 8–15 nucleobases (cstrX, Figure S1).

For all heterodimeric dsDNAs, LILBID mass spectra were measured at varying laser intensities and for each spectrum, the proportions of dissociated DNA (*r*_D,LILBID_) were determined. Each data set contained the *r*_D,LILBID_ value and the correlated droplet explosion width (ew) 5 μs after irradiation (Figure 2A). Plotting *r*_D,LILBID_ against ew yielded dissociation plots similar to those shown in Figure 2B (red plot). For all dsDNAs, we found a linear relationship between r_D, LILBID_ and the ew between 750 and 1250 μm (Figure S2). In this ew range, the laser desorption was strong enough to produce a sufficient ion count but no ions were lost due to the geometry of the ion optics.

**Figure 2:**
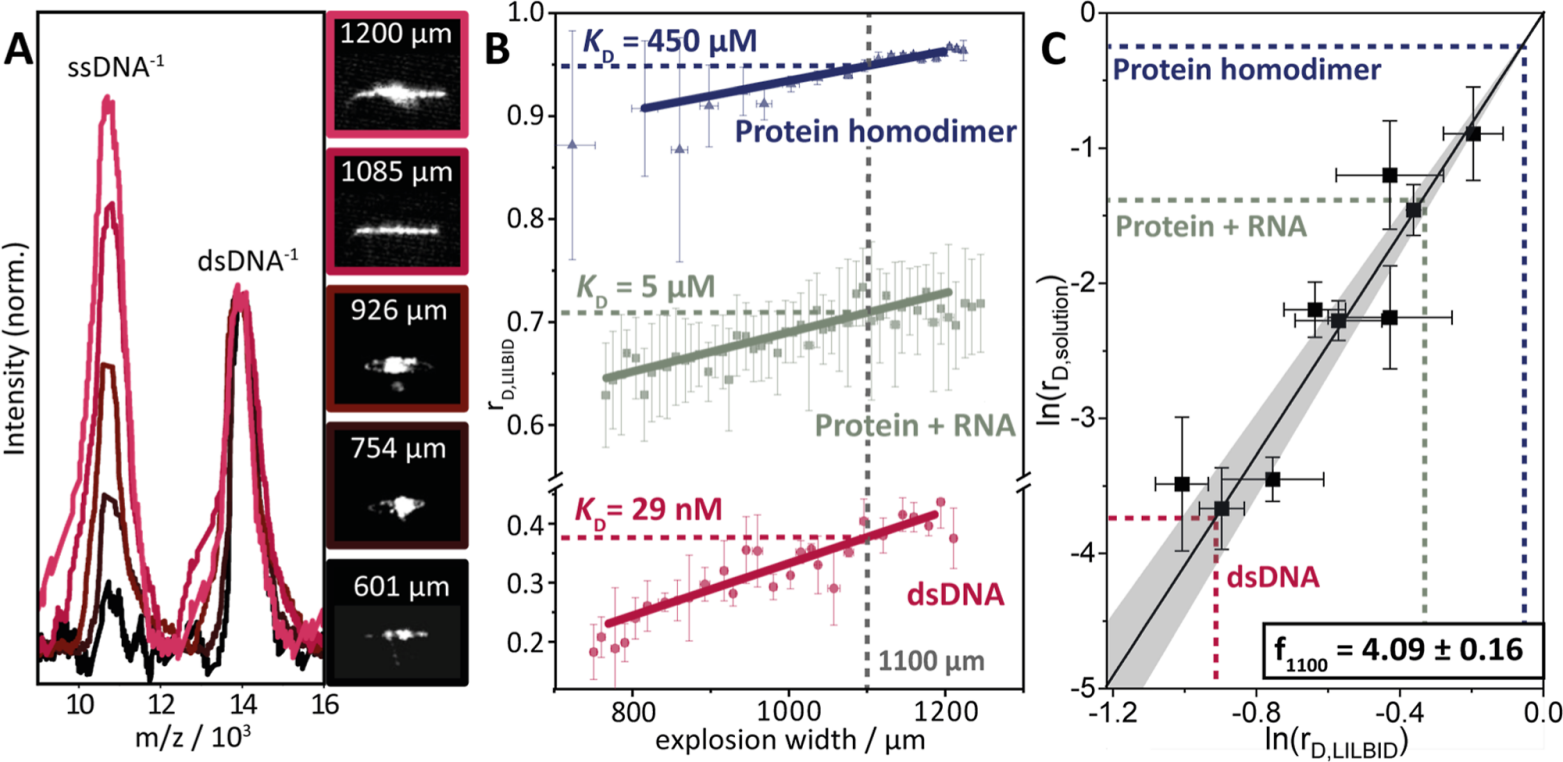
Analysis of LILBID mass spectra and droplet explosions for the *K*_D_ determination of diverse biomolecular dimers using a dsDNA calibration standard. (A) LILBID mass spectra (left) and corresponding images of droplet explosions (right, taken 5 μs post IR irradiation) are shown at different laser energy transfer levels. LILBID mass spectra of dsDNA (strC + cstrC(7–16),^40^ see Figure S1) show enhanced dimer dissociation with increasing droplet explosion width, a proxy for the transferred laser energy into the droplet. The measurements were conducted with 30 μM of each DNA strand, and the LILBID mass spectra were normalized to the height of the dsDNA peak. (B) Exemplary dissociation plots of different biomolecular dimers. The dsDNA analyte contains strC + cstrC(7–16) (see Figure S1 and Table S4), the “protein + RNA” sample contains the RNA binding protein Roquin and a linear Roquin binding element (LBE) composed of an 15 nucleotide ssRNA^35^ (see Table S8), and the “protein homodimer” sample contains the *T. brucei* oxidoreductase Tpx with the covalently bound molecular glue CtFT (see Figure S4 and Table S6).^44^ The degree of dissociation (*r*_D,LILBID_) is plotted against the explosion width (ew), and the *r*_D,LILBID_ at ew = 1100 μm, required for *K*_D_ determination, is indicated with dotted lines. (C) Calibration plot with the logarithm of *r*_D,solution_ plotted against the logarithm of *r*_D,LILBID_. The proportionality factor *f*_1100_ was obtained via linear regression (black line) according to eq 6. The calibration points (black) stem from different calibrant dsDNAs with known *K*_D_s,^40^ and have been measured at 30 μM analyte concentration (see Table S3). The gray area represents the confidence interval of 0.95. The dotted lines are placed according to the *r*_D,LILBID_ values calculated from the exemplary dissociation plots in B. Based on the correlation factor *f*_1100_, the *K*_D_ values of DNA, protein/RNA and protein dimers can be determined by using eqs 4a or 4b, and 6.

For precise calibration, the dissociation behavior of all calibrant dsDNAs had to be assessed at the same explosion width (ew) to ensure that a comparable laser energy transfer occurred in all analyte droplets. Since a large laser energy transfer favors high ion counts and therefore better signal-to-noise ratios, a high explosion width in the linear region should be selected to obtain mass spectra with high reliability. Here, we chose an ew of 1100 μm which showed the highest ion count (see Young et al.^40^) and no loss of ions due to the geometry of the ion optics, which might create a bias to larger *m*/*z*. We determined the r_D,LILBID_ values for all calibrant dsDNAs from their respective, linearly fitted dissociation plots (Figure S2). Figure 2C shows the calibration plot, with the logarithm of these *r*_D,LILBID_ values plotted against the logarithm of the corresponding *r*_D,solution_ values, which were calculated using *K*_D_s from literature with eq S12b.^40^ Finally, for the given set of calibrants and experimental setup, a linear fit yielded the proportionality factor *f*_1100_ = 4.09 ± 0.16 which was used for all following *K*_D_ determinations.

### *K*_D_ Values for Homodimers—Method Validation

In all previous studies that employed qLILBID-MS for *K*_D_ determination, the biomolecular analytes formed heterodimers and had a large mass difference between binding partners. This prevents an overlap in charge states,^35,40,47^ allowing for an unambiguous assignment of peak integrals to the different analyte species. In addition, binding a much smaller interaction partner barely affects the ionization efficiency, and thus the charge state distribution, of the larger monomer which allowed to obtain *r*_D,LILBID_ from the integrals of singly charged MS peaks from the larger monomer and the heterodimeric complex. However, homomeric interactions are very common in biomacromolecules, thus presenting the urgent need to extend the use of qLLIBID-MS also to such systems.

To adapt the qLILBID method for homodimers, we had to consider all charge states of monomer and dimer species, as well as charge state overlaps of e.g. singly charged monomer and doubly charged dimer with identical *m*/*z*. To account for the latter, we pursued two different strategies, cross-linking and dilution, i.e. to either create permanent dimers or to strongly suppress dimerization. Using the well-characterized model protein bovine serum albumin (BSA) with a homodimer *K*_D_ of 10 ± 2 μM as a standard,^48^ we could determine the charge state distributions of both dimer and monomer. Knowing the charge state ratio of either the monomeric or the dimeric species then sufficed to calculate their relative abundance in the overlapping MS peaks.

### Cross-Linking Approach

In LILBID mass spectra recorded with BSA solution at a concentration of 30 μM (Figure 3A, red spectrum), we mainly observed monomers with one to four negative charges, but also smaller peaks that indicated BSA dimers (BSA_2_^1–^, BSA_2_^3–^). From this, we inferred that additional dimer peaks (BSA_2_^2–^, BSA_2_^4–^) overlapped with peaks of the BSA monomer (BSA^1–^, BSA^2–^).

**Figure 3:**
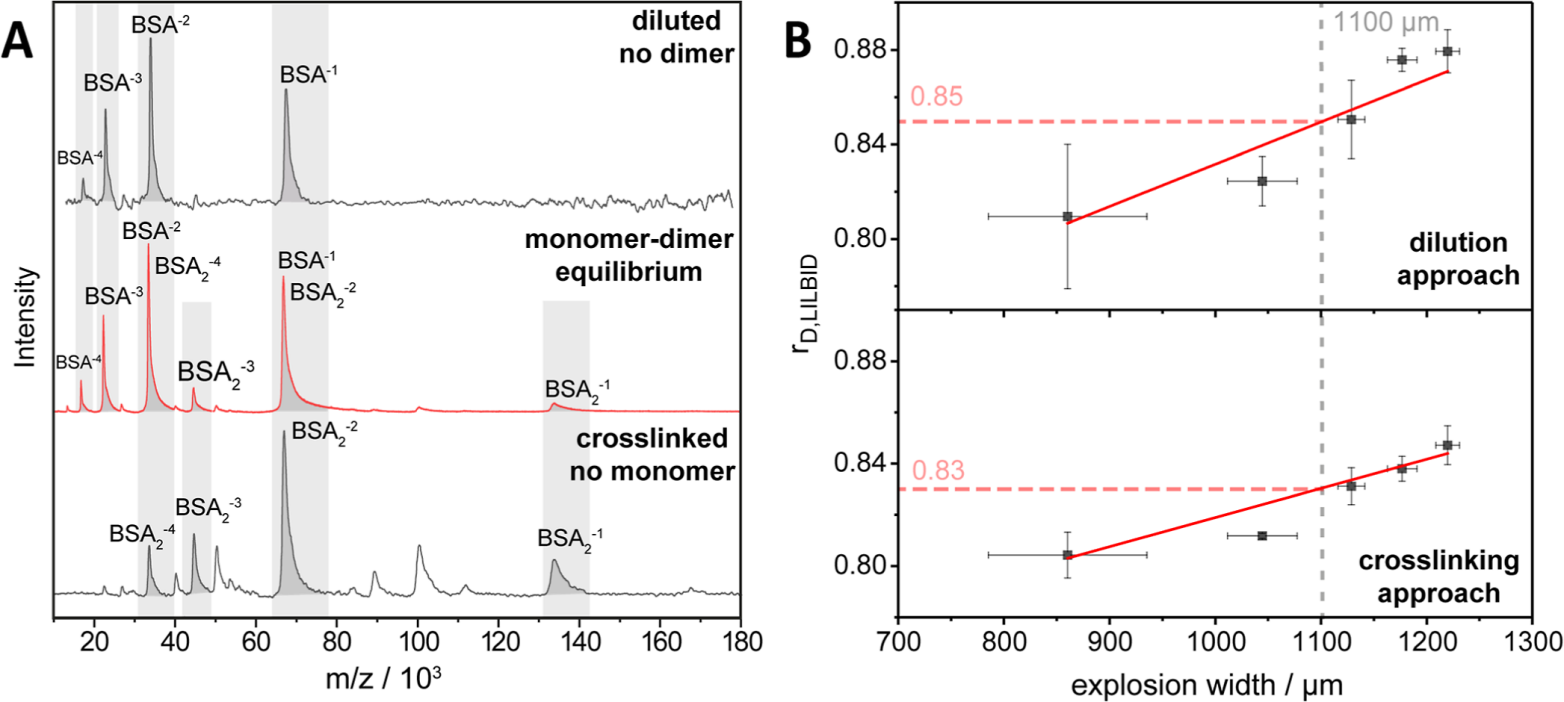
Cross-linking and dilution approaches can be used to disentangle overlapping charge states of protein monomers and dimers. (A) MS-spectra of BSA, diluted (100 nM - top, black), undiluted (30 μM - middle, red) and crosslinked (30 μM - bottom, black). MS-peaks corresponding to BSA monomers and dimers are highlighted in gray. Either the spectra of diluted BSA or of cross-linked BSA dimers can be used to determine the amount of monomer and dimer in the mass spectra of unmodified BSA, and calculate *r*_D,LILBID_ with eq 5. Additional peaks in the cross-linked spectra stem from higher oligomers and do not interfere with the analysis. (B) BSA dissociation plots obtained following the dilution (top) or the cross-linking approach (bottom) to disentangle overlapping MS peaks in the same MS spectrum of 30 μM BSA (A, red). *r*_D,LILBID_ was plotted against the explosion width (see Table S9) and linearly fitted between 750 and 1250 μm to obtain *r*_D,LILBID_ at an ew of 1100 μm for *K*_D_ determination with eqs 4b and 6 (for details, see main text).

To quantify the relative abundances of each species within the overlapping peaks, we analyzed the charge state distribution of isolated dimer signals by cross-linking BSA with the reagent 1-ethyl-3-(3-(dimethylamino)­propyl)­carbodiimide (EDC) and removing monomeric protein via filtration prior to the qLILBID-MS analysis (Figure 3A, bottom).

Next, we determined the relevant peak areas for these charge states (−1 to −4) and scaled this pattern to the “dimer only” peaks observed in the MS spectrum of unmodified BSA (BSA_2_^–1^ and BSA_2_^3–^ at *m*/*z* 132 k and 44 k respectively). Subtracting the scaled BSA dimer contribution revealed the peak intensities attributable to monomers.

As laser energy transfer influences the analyte’s ionization, and thus the charge state distribution of each species, we determined a charge state correlation function cs_corr_(ew) which describes the linear charge state distribution shift in dependence of ew (Figure S3). Importantly, mass spectra of cross-linked and unmodified BSA have to be compared for the same ew (see Supporting Information for details, eqs S1–S9 and Figure S3A,B). We determined the *r*_D,LILBID_ for BSA at different explosion widths between 600 and 1350 μm, and interpolated an *r*_D,LILBID_ of 0.83 ± 0.03 for ew = 1100 μm (Figure 3B, bottom). With the *f*_1100_ value established with the dsDNA calibrants and eq 6, we calculated a *r*_D,solution_ of 0.47 ± 0.07. Using eq 4b and *c*_total_ = 30 μM, we ultimately obtained a BSA dimer *K*_D_ of 8 ± 4 μM which is in good agreement with the literature value of 10 ± 2 μM.^48^

### Dilution Approach

To bias the sample composition to the monomeric state, we analyzed a diluted BSA solution with a concentration of 100 nM. This is well below the reported BSA dimer *K*_D_, and yields less than 1% dimer in solution, thereby allowing the unambiguous and determination of the charge state distribution of BSA monomers (Figure 3A, top). Following this approach, we were able to reliably disentangle the spectral contributions of BSA monomers and dimers at the higher protein concentration (30 μM, Figure 3A, red spectrum).

As for the cross-linking approach, we considered the effect of the laser energy transfer on the analyte’s ionization, and recorded mass spectra of 100 nM BSA at explosion widths between 600 and 1350 μm (Figure S3A). By scaling the charge state distribution of the purely monomeric species based on non-overlapping peaks, similar as previously as done with the cross-linked dimers, we retrieved the separated signal intensities stemming from monomers and dimers, respectively.

This yielded *r*_D,LILBID_ values which were linearly fitted (Figure 3B, top) to obtain an *r*_D,LILBID_ of 0.85 ± 0.02 at an ew of 1100 μm. Using eq 6 and *f*_1100_ for our experimental setup, we calculated an *r*_D,solution_ of 0.52 ± 0.05, which, together with eq 4b and *c*_0_ = 30 μM, yielded a *K*_D_ of 11 ± 4 μM. Again, our approach was in good agreement with the literature value of 10 ± 2 μM.^48^

In summary, both the cross-linking and dilution approach yielded *K*_D_ values (8 ± 4 μM and 11 ± 4 μM, respectively) matching the reported literature value (10 ± 2 μM) for BSA dimerization.^48^ Our results thus show that both strategies can be used to reliably quantify homodimer affinities with LILBID-MS. Overall, the dilution approach was more sample- and time-efficient than cross-linking, as it did not require prior chemical modification. However, the dilution approach is in general only applicable to samples with sufficiently low binding affinities since complex dissociation has to occur at sample concentrations that do not undercut the LILBID detection limit.^41^ This sample dependent detection limit currently lies in the high nM range and hence limits the dilution approach to *K*_D_s larger than 1–10 μM. Further, dilution decreases the signal-to-noise ratio and thus increases the method’s intrinsic error. Therefore, the cross-linking approach is preferable when a sufficient amount of sample is available.

### Determination of Chemically Induced Protein Homodimer Affinities Using qLILBID-MS

Encouraged by the accurate determination of BSA homodimer affinity, we next sought to benchmark qLILBID-MS using a set of chemically inducible protein homodimers. As we previously reported, the homodimerization of the *T. brucei* oxidoreductase Tpx is strictly contingent on the interaction with small molecular glues derived from the covalent inhibitor *para*-CFT (Figure 4A,B).^43,44^

**Figure 4:**
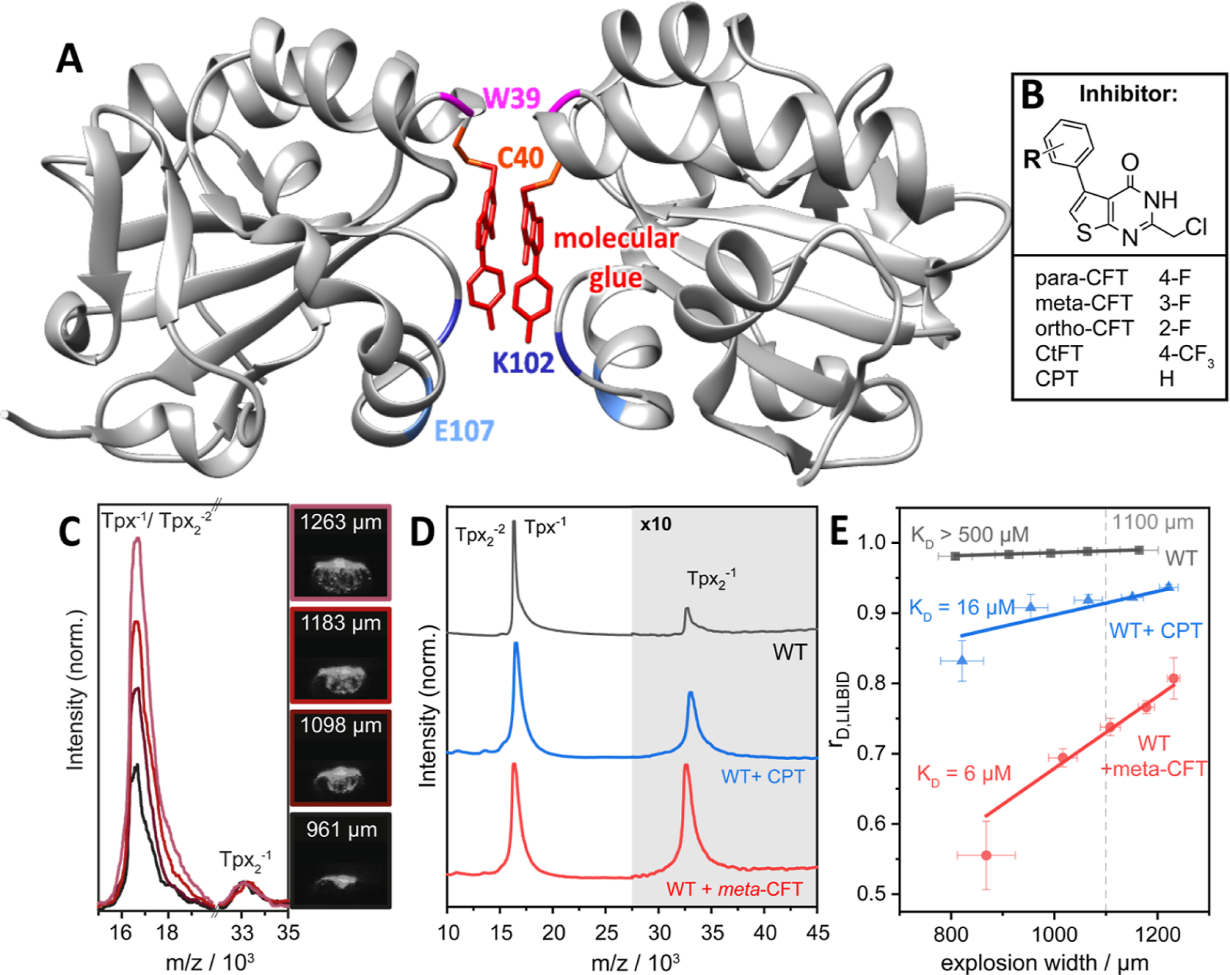
qLILBID-MS accurately captures affinities of chemically induced Tpx homodimers with different molecular glues. (A) X-ray crystal structure of the chemically induced Tpx dimer (PDB: 6GXG, chains A, B).^43^ The dimer interface consists of a molecular glue (here 2-(chloromethyl)-5-(4-fluorophenyl)­thieno­[2,3-*d*]­pyrimidin-4­(3*H*)-one, “para-CFT”) covalently bound to Tpx residue C40 (red), two tryptophane residues (W39, pink), and salt bridges between the charged residues (K102 and E107, dark and light blue, respectively) (all side chains shown in Figure 5). The tertiary structure of the protein backbone is shown in gray. (B) Structural formulas of multiple molecular glues that induce Tpx dimerization upon covalent binding to cysteine residue 40.^44^ (C) LILBID mass spectra of Tpx WT with covalently bound *meta*-CFT (left) and enhanced images of droplet explosions with varying sizes taken 5 μs post IR irradiation (right). The measurements were conducted at a concentration of 30 μM Tpx and the spectra were normalized to the peak height of the dimer peak at *m*/*z* = 33·10^3^. (D) LILBID mass spectra of Tpx WT without inhibitor (top, gray), or with the molecular glues CPT (blue, middle), and *meta*-CFT (red, bottom). For all shown plots 98 spectra were summed, each resulting from one droplet’s explosive expansion. The sample concentration was 30 μM and the area around the dimer peak is displayed with a 10-fold intensity (gray background). (E) Exemplary dissociation plots (see Table S6) of unmodified Tpx WT (gray, top), Tpx WT + CPT (blue, middle), and Tpx WT + *meta*-CFT (red, bottom). The dotted line marks the explosion width of 1100 μm which is used to extrapolate *r*_D,LILBID_ for the *K*_D_ determination. The annotated *K*_D_ is the result of three measurements (Figure S4) and was calculated using eqs 4b and 6.

Binding of these molecular glues to the active site cysteine40 results in the formation of Tpx homodimers with *K*_D_ values in the micromolar range.^43,44^ In addition to the molecular glue (Figures 4A and 5D), the Tpx homodimer interface consists of two tryptophane side chains (W39, Figures 4A and 5D) and two salt bridges (between K102 and E107′ from another chain, Figures 4A and 5A). Point mutations of either of these residues in the dimer interface, and changes in the fluorination pattern of the molecular glue, can tune Tpx homodimer affinity by 2 orders of magnitude in the micromolar range^43,44,46^ (Table 1). Hence, the Tpx-based CID system constitutes an excellent standard to benchmark our qLILBID-MS method.

**Table 1:** *K*_D_ Values of Tpx Homodimers Obtained from qLILBID-MS and Previous ITC Measurements

Tpx	molecular glue	*K*_D,LILBID_ [μM]	*K*_D,ITC_ [μM] ^43,44^
WT	none	>500	-
	*para*-CFT	5 ± 4	5.3 ± 1.9
	*meta*-CFT	6 ± 5	3.7 ± 1.5
	CPT	16 ± 9	6.0 ± 2.2
	*ortho*-CFT	26 ± 13	32 ± 7
	CtFT	360 ± 110	410 ± 56
W39A	none	>500	-
	*para*-CFT	300 ± 110	-
K102A	none	>500	-
	*para*-CFT	36 ± 21	-
K102E	none	310 ± 120	-
	*para*-CFT	70 ± 16	83 ± 32

Due to the sufficient availability of purified protein, we first decided to pursue the cross-linking approach to determine the charge state distribution of cross-linked Tpx homodimers at different explosion widths (Figure S3C). Using the resulting charge state correlation function from the measurement of cross-linked Tpx allowed us to extract the *r*_D,LILBID_ from mass spectra of Tpx samples with mixed monomer/dimer populations.

Next, we analyzed the effect of different molecular glues (*para*-CFT, *meta*-CFT, *ortho*-CFT, CPT, and CtFT, see Figure 4B) on the formation of chemically induced homodimers of Tpx wild type (WT). To this end, we followed the previously established qLILBID workflow (Figures 1 and 4C,D), fitted the resulting dissociation plots (Figures 4E and S4), determined *r*_D,LILBID_ at an ew of 1100 μm, and calculated the Tpx homodimer affinities (*K*_D,LILBID_) using eqs 4b and 6 (Figure 4E and Table 1, Suppoprting Information Tables S2 and S3).

Without molecular glue, Tpx WT shows a very limited propensity to dimerize which manifested in very low or absent dimeric signals, and did not yield an explicit dissociation constant (*K*_D,LILBID_ > 500 μM). By contrast, the addition of all molecular glues induced Tpx homodimers with distinct *K*_D_ values between 5 ± 4 μM (*para*-CFT) and 370 ± 110 μM (CtFT), clearly reflecting their varying affinities. Comparing dissociation constants determined by qLILBID, (*K*_D,LILBID_) with those previously determined by dilution-ITC (*K*_D,ITC_)^43,44^ shows that qLILBID-MS accurately captured Tpx homodimer affinities (Table 1), requiring significantly less time and sample material. This establishes qLILBID as a powerful tool for the analysis of noncovalent protein homodimers over a physiologically relevant affinity range and prompted us to speculate whether our method would also be suited to readily screen the effect of mutations in a dimer interface.

To this end, we mutated Tpx residues W39 and K102 which were previously shown to be crucial constituents of the chemically induced Tpx homodimer interface,^43^ to alanine. Moreover, K102 was mutated to glutamate to analyze the effect of charge repulsion within the dimer interface. The homodimer affinities of all Tpx mutants with and without bound molecular glue were determined using the herein established qLILBID-MS workflow (Figure 5 and Table 1).

**Figure 5:**
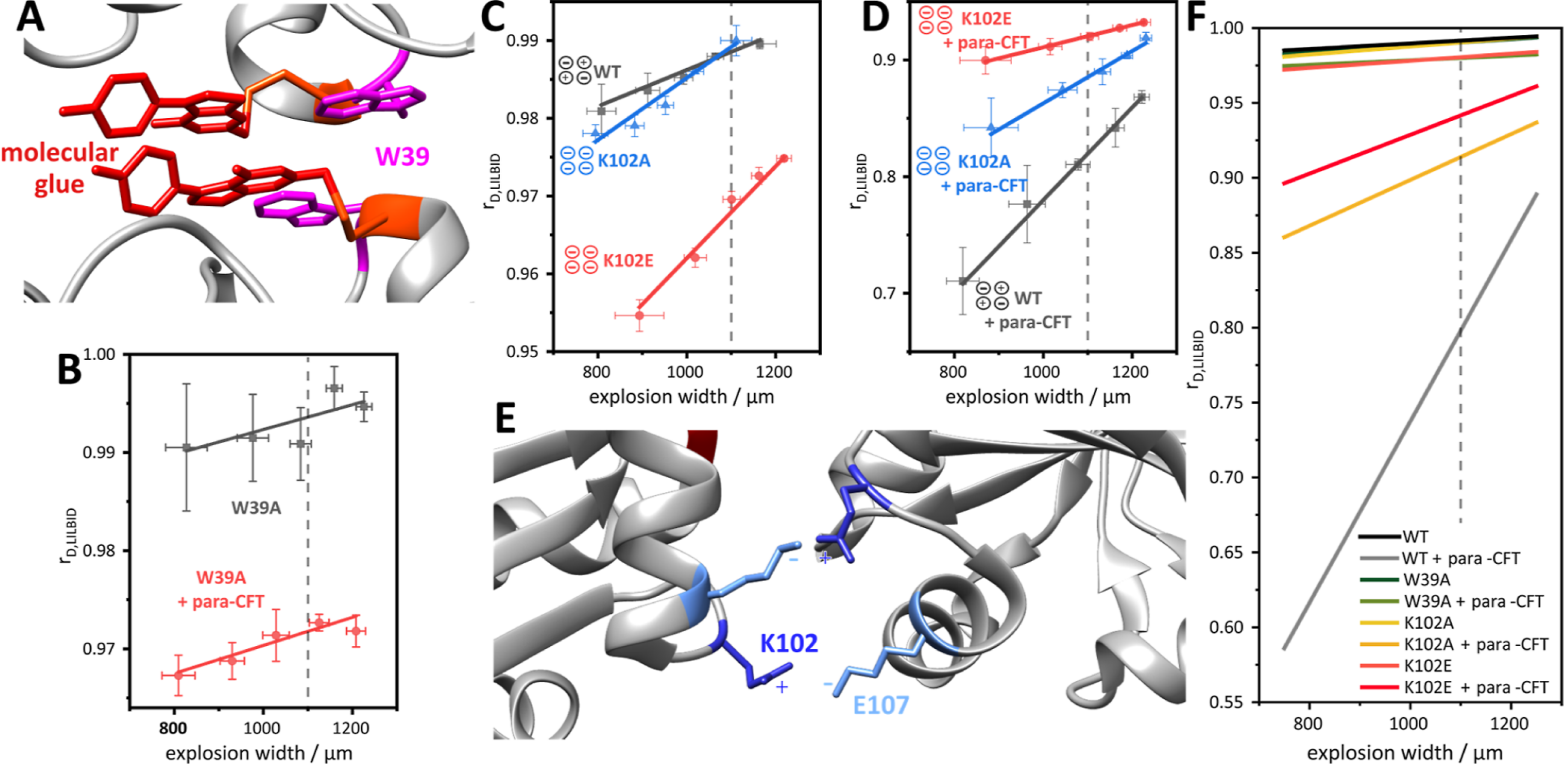
(A) Zoom into the Tpx dimer interface highlighting side chains of Tpx residues W39 from both protomers (pink), which contribute to hydrophobic interactions in the dimer interface. (B) Dissociation plots of Tpx W39A and W39A + para-CFT (see Table S7). Data shown are exemplary results from triplicate measurements (Supporting Information Figures S4 and S5). As expected, removing the indole side chains from the interface greatly reduced homodimer affinity. (C) Dissociation plots of Tpx WT, K102A and K102E (see Table S7). Data shown are exemplary results from triplicate measurements (Supporting Information. Figures S4 and S5). Without inhibitor, all constructs are predominantly monomeric. (D) Dissociation plots of *para*-CFT-bound Tpx WT, K102A and K102E (see Table S7). Data shown are exemplary results from triplicate measurements (Supporting Information Figures S4 and S5). As anticipated, decreasing the charge complementary in the dimer interface leads to a decrease in chemically induced homodimer affinity (WT > K102A > K102E). To determine the respective *K*_D_ values, *r*_D,LILBID_ was determined at an ew of 1100 μm for all species (dotted lines). (E) Zoom into the Tpx dimer interface highlighting side chains of Tpx residues K102 and E107, which form intermolecular salt bridges that stabilize the chemically induced Tpx dimer interface.^43^ (F) Mean dissociation plots of all mutants and the WT with and without *para*-CFT as molecular glue. The dissociation plots are calculated as mean of the fits resulting from the triplicate measurements of each species.

The Tpx W39A mutation was introduced to disrupt key hydrophobic interactions in the dimer interface, as gauged by quantitative studies using SEC, SEC-MALS, SEC-SAXS and ^19^F NMR.^43–46^ Similar to the WT protein, the Tpx W39A mutant was monomeric in solution, however, the affinity of the corresponding, chemically induced homodimers (*K*_D_ = 300 ± 110 μM) was strongly reduced compared to the WT protein (*K*_D_ = 5 ± 4 μM) (Table 1, Figures 5A,B and S5). Since ITC requires sample concentrations well above the *K*_D_, attempts to determine the [Tpx W39A/*para*-CFT]_2_ homodimer affinity with dilution ITC failed previously.^43^ In contrast, qLILBID-MS provided a *K*_D_ value for these low-affinity homodimers, which is in good agreement with estimates from other methods.^43–46^

Next, we turned to residue K102 which engages in ionic interactions with glutamate residue 107′ from another Tpx protomer (Figure 5E). To decrease dimer affinity through the stepwise removal of charge complementarity in the dimer interface, we mutated residue K102 either to alanine (K102A) to remove the salt bridge, or to glutamate (K102E) to create charge repulsion in the dimer interface. While the alanine mutant without molecular glue again had very low dimer affinity like the WT protein (>500 μM), the unmodified Tpx K102E mutant showed an increased homodimer affinity (310 ± 120 μM) which could result from unspecific polar interactions introduced by the mutation (Table 1 and Figure 5C).

After homodimer induction with *para*-CFT, the different degree of charge complementarity in the dimer interfaces of Tpx WT, Tpx K102A and Tpx K102E was reflected in the corresponding homodimer affinities determined with qLILBID-MS. While [Tpx WT/*para*-CFT]_2_ with intact ionic interactions between the two protomers had a *K*_D_ of 5 ± 4 μM, the K102A mutant ([Tpx K102A/*para*-CFT]_2_) lacking these interactions showed a decreased homodimer affinity (*K*_D_ = 37 ± 21 μM). The K102E ([Tpx K102E/*para*-CFT]_2_) mutation further decreased homodimer affinity, possibly due to negative charge repulsion in the dimer interface, and the dissociation constant obtained via qLILBID-MS (*K*_D_ = 71 ± 16 μM) again matched the previous determination using dilution ITC^43^ (Figures 5D, S5 and Table 1).

In summary, we could use qLILBID-MS to determine the dimer *K*_D_s of a diverse set of protein homodimers that vary in their structural composition and dimer affinity in a fast and sample-efficient manner.

## Conclusion

This study demonstrates that qLILBID-MS is an excellent method to determine (induced) protein dimer affinities in physiologically relevant ranges and therefore complements other available methods. Furthermore, we showed that in some cases, such as the chemically induced Tpx dimer, qLILBID can provide results where other methods, such as ITC, fail due to technical limitations. As homodimers present a particular challenge for *K*_D_ analysis with qLILBID-MS, we developed two different strategies, protein cross-linking and sample dilution, to resolve overlapping MS peaks arising from multiple oligomeric species with identical *m*/*z* values. Analyzing BSA and chemically induced Tpx homodimers, we found that both approaches yield *K*_D_ values that are in good agreement with the literature.^43,44,46^ This set of validation methods should allow researchers to assess the affinities of their biomacromolecules of interest in a straightforward manner, even when faced with limited sample amounts or low affinity homodimers.

To test whether qLILBID-MS is a robust method that can detect small variations in dimer affinity, we analyzed a chemically diverse set of induced Tpx homodimers with various molecular glues and affinities for systematic benchmarking. Small chemical modifications of the molecular glue shifted the *K*_D_ value of induced Tpx homodimers by 2 orders of magnitude and, following the herein established protocol, we show that qLILBID-MS accurately reflects our previously reported dimer *K*_D_s.^44^

Tpx dimerization with the molecular glue similarly hinges on the interplay of amino acid side chains in the dimer interface, i.e. W39, K102 and E107.^43^ Importantly, qLILBID was able to determine high *K*_D_ values inaccessible by ITC. In line with the expected structural contributions of the W39 in the Tpx interface, a *K*_D_ shift of almost 2 orders of magnitude compared to Tpx WT was observed. This is in good agreement with estimates from prior studies that employed ^19^F NMR spectroscopy.^44,46^ Our results also reinforce the importance of ionic interactions in the Tpx dimer interface in the presence of molecular glue, explicitly between residues K102 and E107.^43^ By mutating K102 to alanine and glutamate, we progressively disrupted charge complementarity and assessed the impact on homodimer affinity via qLILBID-MS. The K102A mutation eliminated energetically favored salt bridges, which is reflected in a 7-fold decrease in homodimer affinity. Charge inversion achieved with the K102E mutation further destabilized the interface through electrostatic repulsion, causing a more than 10-fold decrease in affinity compared to Tpx wildtype.

In addition to its accuracy and robustness, qLILBID-MS offers rapid analysis of *K*_D_ values for both homo- and heterodimeric biomolecules, while requiring minimal sample consumption. Typical *K*_D_ determination with qLILBID-MS requires 650 pmol of samples for the cross-linking approach (5 μL sample, at 10 μM concentration, three repeats +20 μL sample at 30 μM for the cross-linking process) and ∼50 pmol for the dilution approach (5 μL sample, at 10 μM concentration, three repeats and 5 μL at ∼100 nM).

Importantly, the protein complexes investigated herein exhibited affinities in the micromolar range, which is characteristic of many physiologically relevant protein–protein interactions.^1,49^ Notably, this includes *K*_D_ values in the high micromolar range indicative of weak affinities. While such interactions are often biologically meaningful, e.g. in transient signaling interactions, they are notoriously difficult to quantify using conventional methods like ITC, which require sample concentrations larger than the *K*_D_. Due to this limited accessibility of confirmed high *K*_D_ values, reference data in this range were scarce for the investigated samples, implying that further validation in this range would be beneficial.

Our approach can distinguish between small differences in affinities, driven e.g. by the loss of a salt bridge, or the fluorination pattern of a molecular glue, which is an important prerequisite for dissecting biological interactions or drug development. Together with our previous studies on DNA and protein/RNA heterodimers,^35,40^ this highlights the broad applicability of qLILBID-MS that allows a fast and precise determination of *K*_D_ values for a plethora of biomacromolecular complexes.

Future improvements, such as coupling a LILBID ion source to a commercial mass spectrometer, will allow for state-of-the-art mass resolution. Continued methodological advancements will aim to enable the determination of *K*_D_ values in increasingly complex systems including heterogeneous samples, higher-order oligomeric assemblies, integral membrane proteins, and lipid/protein interactions or determine the influence of small modifications as post translational modifications (PTMs) on affinities. Ideally, these advances will also support high-throughput capabilities, thereby broadening the method’s applicability across diverse areas of structural and functional biology.

## Supplementary Material



## Notes

The data sets generated and analyzed in this paper, together with the Python script used for data interpretation can be accessed at GUDe (Goethe University Data Repository) (10.25716/gude.128z-2wdt) (PDF)
