# Supplementary material for: Quantifying Protein Homodimer Affinities and the Effect of Molecular Glues and Interface Residues Using Native Mass Spectrometry
Source: J Am Chem Soc. 2026 Apr 1;148(14):14813–23. doi: 10.1021/jacs.5c18602 (PMC13088243; doi:10.1021/jacs.5c18602)
Supplement: Supplementary file 1 [file ja5c18602_si_001.pdf]

Supporting Information

Quantifying protein homodimer affinities and the effect of molecular glues and interface residues using native mass spectrometry

Jonathan Schulte‡ [a], Eric Schwegler‡ [b], Ute A. Hellmich[b, c, d], Nina Morgner*[a, e]

[a] Institute of Physical and Theoretical Chemistry
Goethe-University, 60438 Frankfurt/Main,
Max-von-Laue-Str. 9, 60438 Frankfurt/Main, Germany
[b] Faculty of Chemistry and Earth Sciences
Institute of Organic Chemistry and Macromolecular Chemistry,
Friedrich-Schiller-University, 07743 Jena, Germany
[c] Center for Biomolecular Magnetic Resonance (BMRZ)
Goethe-University, 60438 Frankfurt/Main, Germany
[d] Cluster of Excellence Balance of the Microverse
Friedrich-Schiller-University, 07743 Jena, Germany
[e] Cluster of Excellence SubCellular Architecture of Life (SCALE),
Goethe-University, 60438 Frankfurt/Main, Germany

‡shared first authors

*for correspondence: morgner@chemie.uni-frankfurt.de

Table of Contents

Table S1 .... S-2
Table S2 .... S-4
Table S3 .... S-5
Table S4 .... S-6
Table S5 .... S-10
Table S6 .... S-11
Table S7 .... S-13
Table S8 .... S-15
Figure S1 .... S-17
Figure S2 .... S-18
Figure S3 .... S-19
Figure S4 .... S-20
Figure S5 .... S-21
Figure S6 .... S-22
Calculation of $r_{D,LILBID}$ from peak integrals S-23
Derivation of K_Ds from $r_{D,solution}$ S-24
Materials and Methods S-26
References S-28

Table S1: Linear fits from dissociation plots for the affinity calibration.

Fitting parameters obtained from the qLILBID calibration using the dsDNA calibration set (**Figure S1**). For each set of binding partners, the monomer-to-complex ratio ($r_{D,LILBID}$, equation S9) was extracted from the mass spectra across a range of explosion widths and a linear fit was applied (**Figure S2**). From these fits, $r_{D,LILBID}$ was determined for an explosion width of 1100 µm. Available K_Ds from the literature[1] were used to calculate $r_{D,solution}$ values (equation (S3)). Plotting $r_{D,solution}$ against $r_{D,LILBID}$ yields a calibration plot (**Figure 2C**), which was used to determine K_D values throughout this study.

binding partners	fit - slope	fit - y-intercept	$r_{D,LILBID}$ @ 1100 µm	$K_{D,ITC}$ / nM[1]	$r_{D,solution}$
**strA + cstrA(16-26)**	8.07E-04 ± 1.71E-04<br>1.02E-03 ± 3.63E-04<br>4.58E-04 ± 3.36E-05	-0.081 ± 0.171<br>-0.586 ± 0.372<br>0.112 ± 0.035	**0.653 ± 0.12**	370 ± 50	**0.45 ± 0.02**
**strA + cstrA(18-26)**	2.59E-04 ± 1.03E-04<br>1.75E-04 ± 8.50E-05<br>2.59E-04 ± 3.96E-05	0.627 ± 0.101<br>0.533 ± 0.084<br>0.627 ± 0.038	**0.824 ± 0.077**	8500 ± 700	**0.904 ± 0.007**
**strA + cstrA(21-35)**	5.90E-04 ± 7.60E-05<br>4.02E-04 ± 6.49E-05<br>5.02E-04 ± 3.35E-05	-0.070 ± 0.074<br>-0.052 ± 0.067<br>-0.103 ± 0.034	**0.473 ± 0.079**	31.2 ± 0.8	**0.162 ± 0.002**
**strA + cstrA(26-35)**	1.02E-04 ± 3.03E-04<br>4.11E-04 ± 2.68E-04<br>7.71E-04 ± 7.63E-05	0.364 ± 0.296<br>0.175 ± 0.272<br>0.007 ± 0.078	**0.653 ± 0.156**	3900 ± 500	**0.83 ± 0.02**
**strB + cstrB(5-16)**	6.97E-04 ± 1.50E-04<br>1.10E-03 ± 3.43E-04<br>2.92E-04 ± 1.76E-04	-0.400 ± 0.150<br>-0.770 ± 0.330<br>0.104 ± 0.170	**0.411 ± 0.031**	20.1 ± 1.8	**0.132 ± 0.005**
**strB + cstrB(6-15)**	5.98E-04 ± 1.52E-04<br>1.02E-03 ± 1.44E-04<br>4.71E-04 ± 1.15E-04	-0.123 ± 0.149<br>-0.409 ± 0.146<br>0.015 ± 0.114	**0.594 ± 0.085**	353 ± 7	**0.443 ± 0.003**
**strB + cstrB(8-16)**	3.15E-04 ± 4.58E-05<br>4.43E-04 ± 2.66E-05<br>2.99E-04 ± 7.94E-05	0.335 ± 0.045<br>0.221 ± 0.026<br>0.366 ± 0.078	**0.695 ± 0.011**	2120 ± 60	**0.741 ± 0.004**
**strC + cstrC(7-16)**	4.61E-04 ± 9.12E-05<br>4.73E-04 ± 9.11E-05<br>4.17E-04 ± 4.86E-05	-0.179 ± 0.088<br>-0.117 ± 0.090<br>-0.091 ± 0.049	**0.366 ± 0.030**	29 ± 7	**0.16 ± 0.02**

**strC + cstrC(8-15)**	2.51E-04 ± 8.75E-05<br>7.39E-04 ± 1.82E-04<br>2.23E-04 ± 7.42E-05	0.242 ± 0.088<br>-0.208 ± 0.178<br>0.229 ± 0.072	**0.431 ± 0.017**	418 ± 16	**0.470 ± 0.006**

Table S2: Linear fits from dissociation plots of Tpx wild type with and without different molecular glues.

Fitting parameters obtained from the qLILBID measurements. For Tpx WT (with or without molecular glue), the monomer-to-complex ratio ($r_{D,LILBID}$, equation (S9)) was extracted from the mass spectra across a range of explosion width and a linear fit was applied (**Figure S4**). From these fits, $r_{D,LILBID}$ was determined for an explosion widths of 1100 µm. The fit from calibration plot (**Figure 2C**) allowed to determine $r_{D,\,solution}$ with equation (6) and therewith $K_{D,LILBID}$ with equation (S16c). K_D Values in the range for which we had reference molecules with known affinities from ITC measurements are printed in black, those values outside of the ITC-validated range are printed in grey.

Tpx WT + molecular glue	fit - slope	fit - y-intercept	$r_{D,LILBID}$ @ 1100 µm	$r_{D,solution}$	$K_{D,LILBID}$ / µM
**WT**	2.01E-05 ± 6.78E-06<br>2.34E-05 ± 4.28E-06<br>1.27E-05 ± 2.12E-06	0.969 ± 0.007<br>0.963 ± 0.005<br>0.981 ± 0.002	0.992 ± 0.003	0.966 ± 0.011	**800 ± 270**
**WT + para-CFT**	9.66E-04 ± 1.55E-04<br>4.54E-04 ± 1.51E-05<br>3.97E-04 ± 9.83E-06	-0.332 ± 0.186<br>0.344 ± 0.017<br>0.383 ± 0.011	0.798 ± 0.048	0.398 ± 0.099	**5 ± 4**
**WT + meta-CFT**	1.15E-04 ± 4.58E-05<br>4.49E-04 ± 1.38E-04<br>5.12E-04 ± 6.67E-05	0.742 ± 0.053<br>0.333 ± 0.151<br>0.167 ± 0.075	0.809 ± 0.058	0.420 ± 0.124	**6 ± 5**
**WT + ortho-CFT**	4.38E-04 ± 4.04E-05<br>-1.43E-05 ± 4.22E-05<br>3.32E-04 ± 1.48E-04	0.381 ± 0.047<br>0.927 ± 0.051<br>0.552 ± 0.171	0.897 ± 0.024	0.642 ± 0.072	**25 ± 13**
**WT + CPT**	1.67E-04 ± 4.76E-05<br>4.95E-04 ± 8.56E-05<br>3.75E-05 ± 7.78E-05	0.731 ± 0.055<br>0.290 ± 0.098<br>0.822 ± 0.078	0.871 ± 0.033	0.568 ± 0.088	**16 ± 9**
**WT + CtFT**	5.20E-05 ± 7.14E-06<br>7.14E-05 ± 4.93E-06<br>5.13E-05 ± 1.69E-05	0.922 ± 0.008<br>0.910 ± 0.005<br>0.924 ± 0.019	0.983 ± 0.004	0.932 ± 0.017	**360 ± 110**

Table S3: Linear fits from dissociation plots of Tpx mutants with and withot para-CFT

Fitting parameters obtained from the qLILBID measurements. For each Tpx variant (with or without molecular glue), the monomer-to-complex ratio ($r_{D,LILBID}$, equation S9) was extracted from the mass spectra across a range of explosion widths and a linear fit was applied (**Figure S5**). From these fits, $r_{D,LILBID}$ was determined for an explosion width of 1100 µm. The fit from calibration plot (**Figure 2C**) allowed to determine $r_{D,solution}$ with equation (6) and therewith $K_{D,LILBID}$ with equation (S16c). K_D Values in the range for which we had reference molecules with known affinities from ITC measurements are printed in black, those values outside of the ITC-validated range are printed in grey..

**Tpx variant + molecular glue**	**fit - slope**	**fit - y-intercept**	**$r_{D,LILBID}$ @ 1100 µm**	**$r_{D,solution}$**	**$K_{D,LILBID}$ / µM**
**K102A**	3.99E-05 ± 5.31E-06<br>3.92E-06 ± 2.26E-06<br>3.70E-05 ± 3.84E-06	0.945 ± 0.005<br>0.988 ± 0.003<br>0.948 ± 0.005	0.990 ± 0.002	0.961 ± 0.007	**690 ± 140**
**K102A + para-CFT**	1.41E-04 ± 1.67E-05<br>9.40E-05 ± 6.35E-06<br>2.24E-04 ± 2.33E-05	0.751 ± 0.019<br>0.847 ± 0.007<br>0.639 ± 0.027	0.914 ± 0.027	0.692 ± 0.084	**36 ± 21**
**K102E**	1.90E-05 ± 8.58E-06<br>2.73E-06 ± 5.40E-06<br>4.95E-05 ± 2.52E-06	0.951 ± 0.010<br>0.983 ± 0.005<br>0.930 ± 0.003	0.981 ± 0.006	0.923 ± 0.025	**310 ± 120**
**K102E + para-CFT**	8.86E-05 ± 9.18E-06<br>1.72E-04 ± 2.11E-05<br>1.28E-04 ± 1.40E-05	0.853 ± 0.011<br>0.741 ± 0.026<br>0.803 ± 0.016	0.942 ± 0.008	0.782 ± 0.029	**70 ± 16**
**W39A**	1.43E-05 ± 3.82E-06<br>5.73E-06 ± 6.41E-06<br>2.56E-05 ± 1.16E-05	0.956 ± 0.004<br>0.979 ± 0.007<br>0.955 ± 0.014	0.990 ± 0.004	0.961 ± 0.016	**680 ± 310**
**W39A + para-CFT**	1.05E-04 ± 1.01E-05<br>1.70E-04 ± 2.66E-05<br>4.05E-05 ± 2.19E-05	0.848 ± 0.012<br>0.754 ± 0.030<br>0.908 ± 0.025	0.980 ± 0.006	0.921 ± 0.023	**300 ± 110**

Table S4: Values for $r_{D,LILBID}$ and the corresponding explosion widths resulting from measurements of calibration DNAs.

strA + cstrA(16-26)											
ew / µm	Δew / µm	$r_{D,LILBID}$	$\Delta r_{D,LILBID}$	ew / µm	Δew / µm	$r_{D,LILBID}$	$\Delta r_{D,LILBID}$	ew / µm	Δew / µm	$r_{D,LILBID}$	$\Delta r_{D,LILBID}$
797.5	14.0	0.151	0.213	772.7	5.5	0.629	0.063	780.9	12.0	0.461	0.003
867.8	21.0	0.420	0.035	813.4	12.1	0.427	0.303	816.1	6.8	0.467	0.033
945.2	36.4	0.186	0.205	871.3	17.9	0.640	0.085	851.5	11.1	0.504	0.027
1024.2	18.9	0.630	0.044	924.6	9.8	0.698	0.055	911.6	16.6	0.527	0.019
1078.9	16.5	0.546	0.056	975.3	20.0	0.735	0.087	951.1	11.3	0.556	0.025
1157.7	30.2	0.622	0.043	1026.3	11.2	0.779	0.063	1000.8	7.5	0.575	0.007
1235.4	18.7	0.583	0.032	1062.2	6.7	0.713	0.084	1031.2	9.3	0.589	0.016
				1089.7	5.3	0.789	0.084	1062.8	5.6	0.587	0.006
				1125.2	17.3	0.877	0.101	1078.7	5.9	0.634	0.007
				1186.4	20.9	0.849	0.068	1096.0	5.8	0.628	0.024
								1114.1	8.1	0.620	0.010
								1145.4	9.1	0.670	0.035
								1171.9	6.6	0.630	0.010
								1200.7	8.6	0.647	0.019
								1236.3	6.7	0.650	0.001
**strA + cstrA(18-26)**											
ew / µm	Δew / µm	$r_{D,LILBID}$	$\Delta r_{D,LILBID}$	ew / µm	Δew / µm	$r_{D,LILBID}$	$\Delta r_{D,LILBID}$	ew / µm	Δew / µm	$r_{D,LILBID}$	$\Delta r_{D,LILBID}$
763.4	9.8	0.734	0.123	763.8	7.6	0.911	0.078	760.5	9.6	0.744	0.004
798.2	17.5	0.639	0.008	820.8	17.3	0.766	0.075	810.6	10.5	0.759	0.020
852.8	16.7	0.687	0.092	863.0	9.3	0.826	0.053	855.9	15.3	0.792	0.049
908.7	12.5	0.680	0.031	887.2	5.2	0.823	0.092	918.1	11.3	0.766	0.037
947.0	3.9	0.663	0.020	913.0	11.1	0.901	0.091	1014.3	45.8	0.827	0.045
1000.4	33.0	0.706	0.015	948.1	10.9	0.907	0.068	1124.2	16.7	0.826	0.043
1087.2	21.0	0.718	0.044	993.5	13.2	0.867	0.102	1209.6	26.3	0.868	0.037
1163.1	22.9	0.709	0.038	1029.5	7.6	0.872	0.091				
1230.0	16.9	0.800	0.070	1068.9	19.8	0.891	0.082				
				1151.9	14.2	0.944	0.079				
				1218.7	18.4	0.959	0.058				

strA + cstrA(21-35)

ew / µm	Δew / µm	$r_{D,LILBID}$	$\Delta r_{D,LILBID}$	ew / µm	Δew / µm	$r_{D,LILBID}$	$\Delta r_{D,LILBID}$	ew / µm	Δew / µm	$r_{D,LILBID}$	$\Delta r_{D,LILBID}$
769.5	10.9	0.403	0.034	772.0	10.5	0.224	0.042	776.1	17.0	0.263	0.041
789.3	5.1	0.373	0.017	813.9	7.4	0.248	0.058	816.3	8.3	0.285	0.020
807.7	4.1	0.415	0.010	853.6	14.1	0.244	0.032	839.0	7.7	0.310	0.014
826.2	9.1	0.426	0.052	899.1	6.5	0.373	0.037	869.0	8.1	0.315	0.018
869.8	11.8	0.448	0.076	923.5	10.9	0.377	0.017	896.8	9.6	0.364	0.034
894.7	7.1	0.452	0.031	958.5	8.5	0.354	0.063	922.8	8.9	0.359	0.028
928.8	13.9	0.508	0.048	997.6	14.2	0.330	0.044	962.7	10.1	0.404	0.037
973.5	8.9	0.493	0.057	1031.4	9.1	0.367	0.021	997.2	7.7	0.429	0.026
1015.7	10.9	0.469	0.049	1071.9	5.8	0.376	0.041	1025.0	6.8	0.442	0.007
1057.9	10.6	0.628	0.085	1093.2	7.1	0.382	0.021	1050.0	8.1	0.431	0.018
1096.6	9.7	0.517	0.007	1116.9	7.9	0.443	0.043	1076.8	7.0	0.447	0.003
1142.7	7.0	0.599	0.048	1164.4	12.6	0.418	0.035	1095.2	5.6	0.459	0.023
1170.5	6.7	0.584	0.069	1187.7	5.5	0.399	0.013	1111.4	5.3	0.457	0.016
1214.2	12.8	0.711	0.153	1204.4	6.4	0.390	0.061	1124.5	6.6	0.458	0.016
				1242.6	10.0	0.447	0.034	1153.0	6.2	0.472	0.017
								1178.9	7.4	0.470	0.004
								1198.5	5.8	0.478	0.014
								1229.6	6.9	0.491	0.028

strA + cstrA(26-35)

ew / µm	Δew / µm	$r_{D,LILBID}$	$\Delta r_{D,LILBID}$	ew / µm	Δew / µm	$r_{D,LILBID}$	$\Delta r_{D,LILBID}$	ew / µm	Δew / µm	$r_{D,LILBID}$	$\Delta r_{D,LILBID}$
761.2	8.0	0.230	0.167	773.1	9.5	0.614	0.065	770.3	11.9	0.667	0.039
817.7	17.6	0.521	0.160	810.9	19.8	0.387	0.275	805.8	11.7	0.588	0.012
866.8	8.1	0.530	0.129	877.6	18.5	0.463	0.095	845.4	9.3	0.662	0.061
910.7	15.2	0.455	0.085	923.6	12.9	0.673	0.037	877.3	9.5	0.679	0.012
977.9	20.7	0.619	0.140	1002.5	18.3	0.704	0.150	909.6	6.2	0.771	0.072
1055.3	26.3	0.536	0.087	1042.5	6.5	0.376	0.126	935.0	7.1	0.673	0.016
1140.4	23.7	0.392	0.096	1078.0	8.1	0.590	0.015	972.5	19.4	0.724	0.045
1212.5	27.8	0.419	0.202	1128.5	17.6	0.647	0.003	1030.3	5.2	0.762	0.038
				1181.0	11.3	0.758	0.118	1059.1	6.5	0.796	0.058
				1229.5	8.8	0.669	0.158	1094.4	17.1	0.805	0.028
								1122.5	3.3	0.917	0.061
								1148.9	12.1	0.942	0.030
								1175.4	6.3	0.936	0.052
								1214.5	12.6	0.966	0.025
								1240.2	4.6	0.937	0.010

strB + cstrB(5-16)

ew / µm	Δew / µm	$r_{D,LILBID}$	$\Delta r_{D,LILBID}$	ew / µm	Δew / µm	$r_{D,LILBID}$	$\Delta r_{D,LILBID}$	ew / µm	Δew / µm	$r_{D,LILBID}$	$\Delta r_{D,LILBID}$
769.3	10.5	0.060	0.049	784.3	9.7	0.105	0.149	764.9	6.0	0.220	0.164
795.0	9.4	0.091	0.129	835.4	17.7	0.043	0.061	795.4	3.4	0.334	0.024
834.2	12.8	0.229	0.056	939.4	49.5	0.390	0.084	825.3	13.2	0.398	0.033
907.0	19.9	0.255	0.115	1045.1	17.9	0.420	0.042	854.2	5.9	0.384	0.085
961.6	11.4	0.368	0.028	1156.2	38.3	0.443	0.034	910.0	18.5	0.398	0.056
1026.2	19.4	0.358	0.059					946.5	6.1	0.319	0.206
1073.3	13.0	0.330	0.065					970.4	10.2	0.484	0.025
1127.4	15.5	0.425	0.016					1024.4	12.1	0.389	0.030
1170.4	12.5	0.452	0.056					1092.2	20.7	0.389	0.104
1226.4	21.0	0.332	0.109					1147.9	10.9	0.567	0.030
								1214.5	21.9	0.341	0.025

strB + cstrB(6-15)

ew / µm	Δew / µm	$r_{D,LILBID}$	$\Delta r_{D,LILBID}$	ew / µm	Δew / µm	$r_{D,LILBID}$	$\Delta r_{D,LILBID}$	ew / µm	Δew / µm	$r_{D,LILBID}$	$\Delta r_{D,LILBID}$
785.5	16.7	0.286	0.041	775.9	19.8	0.436	0.036	767.5	10.8	0.392	0.054
833.0	9.7	0.330	0.052	835.6	15.7	0.409	0.087	811.5	14.2	0.346	0.048
868.8	9.9	0.392	0.020	888.9	19.8	0.449	0.040	851.4	12.9	0.350	0.040
902.4	11.1	0.425	0.063	951.5	4.1	0.608	0.263	891.4	6.9	0.444	0.073
941.4	8.2	0.511	0.017	1000.2	20.9	0.571	0.034	925.5	12.5	0.496	0.079
989.7	10.3	0.554	0.117	1055.7	6.1	0.653	0.101	983.0	10.7	0.519	0.090
1024.9	6.8	0.490	0.056	1085.7	9.3	0.659	0.165	1018.2	8.6	0.473	0.061
1077.2	19.9	0.574	0.020	1122.1	7.0	0.859	0.121	1049.5	12.1	0.611	0.014
1151.2	7.9	0.555	0.072	1154.1	11.1	0.789	0.158	1096.8	13.3	0.530	0.050
1187.5	10.5	0.492	0.021	1206.6	25.5	0.770	0.169	1163.6	10.5	0.547	0.021
								1222.3	17.9	0.534	0.066

strB + cstrB(8-16)

ew / µm	Δew / µm	$r_{D,LILBID}$	$\Delta r_{D,LILBID}$	ew / µm	Δew / µm	$r_{D,LILBID}$	$\Delta r_{D,LILBID}$	ew / µm	Δew / µm	$r_{D,LILBID}$	$\Delta r_{D,LILBID}$
772.8	7.6	0.567	0.017	783.8	19.5	0.567	0.020	773.3	13.9	0.594	0.035
803.1	9.3	0.579	0.056	871.1	28.1	0.614	0.036	835.7	19.9	0.599	0.054
879.5	13.7	0.624	0.025	995.9	45.1	0.656	0.016	890.4	12.1	0.621	0.007
927.3	11.6	0.630	0.054	1160.2	47.2	0.738	0.002	978.7	33.1	0.689	0.024
993.8	24.3	0.678	0.055					1106.0	29.2	0.731	0.053
1066.2	19.1	0.643	0.053					1224.3	25.3	0.698	0.017
1157.2	27.5	0.713	0.004								
1227.2	8.8	0.709	0.005								

strC + cstrC(7-16)

ew / µm	Δew / µm	$r_{D,LILBID}$	$\Delta r_{D,LILBID}$	ew / µm	Δew / µm	$r_{D,LILBID}$	$\Delta r_{D,LILBID}$	ew / µm	Δew / µm	$r_{D,LILBID}$	$\Delta r_{D,LILBID}$
767.9	12.3	0.173	0.082	764.2	10.5	0.191	0.019	769.5	13.2	0.241	0.051
858.9	32.4	0.227	0.024	800.9	9.0	0.270	0.021	802.2	7.1	0.201	0.033
951.9	22.8	0.277	0.020	854.5	16.8	0.322	0.038	837.9	12.6	0.268	0.017
1035.2	23.9	0.258	0.026	909.4	15.5	0.327	0.027	868.8	7.1	0.244	0.032
1162.9	36.5	0.375	0.035	949.3	7.0	0.393	0.060	909.4	16.3	0.305	0.040
				999.4	23.6	0.338	0.012	951.5	17.5	0.313	0.012
				1058.2	14.3	0.319	0.030	996.8	8.6	0.314	0.034
				1099.3	12.7	0.407	0.048	1022.3	4.9	0.361	0.019
				1144.1	16.2	0.412	0.036	1037.7	6.5	0.371	0.018
				1202.8	17.3	0.470	0.067	1087.2	12.5	0.372	0.008
								1113.5	7.3	0.372	0.011
								1153.0	7.4	0.410	0.024
								1186.7	7.0	0.405	0.032
								1223.2	14.9	0.367	0.046

strC + cstrC(8-15)

ew / µm	Δew / µm	$r_{D,LILBID}$	$\Delta r_{D,LILBID}$	ew / µm	Δew / µm	$r_{D,LILBID}$	$\Delta r_{D,LILBID}$	ew / µm	Δew / µm	$r_{D,LILBID}$	$\Delta r_{D,LILBID}$
774.2	12.6	0.466	0.218	797.7	10.3	0.364	0.049	768.2	12.9	0.383	0.018
809.1	14.3	0.484	0.049	845.7	21.1	0.445	0.042	809.5	10.3	0.427	0.030
878.5	15.9	0.398	0.016	964.1	26.1	0.448	0.025	841.4	5.6	0.375	0.049
927.2	10.0	0.482	0.044	1048.4	23.2	0.635	0.210	885.2	6.0	0.462	0.024
986.7	29.8	0.431	0.019	1182.1	23.1	0.640	0.082	930.1	20.1	0.450	0.045
1069.2	22.9	0.529	0.037					991.0	20.5	0.448	0.031
1126.3	16.7	0.542	0.045					1053.8	13.6	0.446	0.023
1163.4	10.8	0.539	0.026					1109.1	15.8	0.517	0.028
1213.6	12.1	0.556	0.032					1201.0	33.9	0.468	0.034

Table S5: Values for peak ratios and the corresponding explosion widths resulting from measurements of crosslinked BSA, crosslinked Tpx-WT and from diluted BSA.

BSA diluted					
ew / µm	Δew / µm	BSA^{-1} / BSA^{-3}	ΔBSA^{-1} / BSA^{-3}	BSA^{-2} / BSA^{-3}	ΔBSA^{-2} / BSA^{-3}
590.9	8.3	0.373	0.285	0.763	0.158
652.1	6.1	1.350	0.854	1.826	1.371
714.0	25.6	1.058	0.458	1.211	0.463
774.7	20.2	1.351	0.113	1.564	0.398
826.8	13.4	2.211	1.650	2.308	0.987
864.4	11.6	1.592	0.530	1.451	0.578
925.8	16.2	1.962	1.189	2.276	1.554
958.5	11.3	0.967	0.611	1.556	0.593
1001.1	12.1	1.656	0.436	2.448	0.958
1018.9	6.8	1.390	0.608	2.095	0.691
1054.3	8.2	1.288	0.296	1.998	0.683
1080.0	10.9	2.088	0.531	2.199	0.595
1110.7	10.9	1.607	0.566	1.855	0.536
1155.6	7.0	2.043	0.938	2.475	1.067
1184.9	19.2	1.517	0.576	1.988	0.787

BSA crosslinked					
ew / µm	Δew / µm	BSA_2^{-2} / BSA_2^{-1}	Δ BSA_2^{-2} / BSA_2^{-1}	BSA_2^{-4} / BSA_2^{-1}	Δ BSA_2^{-4} / BSA_2^{-1}
824.8	76.1	5.154	2.876	5.373	5.199
951.9	8.9	10.291	3.932	3.997	1.866
1060.7	37.3	5.338	2.091	2.535	0.977
1118.1	8.4	3.429	2.020	1.581	1.218
1146.8	6.1	5.094	3.445	1.863	1.055
1169.1	6.2	5.384	3.500	1.761	1.061
1185.3	4.6	3.435	1.390	2.515	1.871
1197.2	3.7	3.176	1.534	0.858	0.475
1208.5	1.8	3.691	2.106	1.543	0.854
1217.1	3.0	4.052	2.657	1.235	0.789
1229.7	3.1	3.877	1.840	1.157	0.711
1240.8	3.2	3.885	2.077	1.185	0.854
1249.1	2.2	2.883	1.445	1.384	0.935
1257.8	2.5	4.109	1.953	1.357	0.957
1265.1	1.7	4.939	2.311	1.608	1.280
1271.1	1.5	3.927	2.510	1.531	0.928
1279.4	3.1	4.848	2.331	1.285	0.561
1288.5	2.3	4.093	1.863	1.718	0.993
1295.5	2.0	4.050	1.862	1.057	0.788
1303.4	2.5	3.740	1.304	1.670	1.197

Tpx-WT crosslinked			
ew / µm	Δew / µm	Tpx^{-1} / Tpx^{-3}	ΔTpx^{-1} / Tpx^{-3}
855.1	19.9	1.073	0.173
897.4	10.0	1.165	0.271
949.1	15.3	1.436	0.479
1018.1	24.9	0.992	0.070
1061.4	7.5	0.688	0.244
1086.5	5.3	0.938	0.025
1125.3	18.1	0.976	0.217
1167.8	8.3	0.889	0.088
1194.0	6.8	0.695	0.243
1211.8	4.7	0.711	0.103
1230.8	6.1	0.819	0.036
1256.8	8.3	0.862	0.120
1281.5	4.5	0.856	0.039
1300.7	5.7	0.831	0.058
1323.7	5.0	0.675	0.127
1334.4	3.9	0.665	0.090

Table S6: Values for $r_{D,LILBID}$ and the corresponding explosion widths resulting from Tpx-WT measurements with and without different covalent inhibitors.

Tpx-WT

ew / µm	Δew / µm	$r_{D,LILBID}$	$\Delta r_{D,LILBID}$	ew / µm	Δew / µm	$r_{D,LILBID}$	$\Delta r_{D,LILBID}$	ew / µm	Δew / µm	$r_{D,LILBID}$	$\Delta r_{D,LILBID}$
785.9	22.2	0.983	0.002	807.9	32.6	0.981	0.003	929.1	55.1	0.992	0.001
871.1	19.5	0.985	0.003	912.2	26.9	0.984	0.002	1033.7	16.2	0.994	0.001
941.0	19.9	0.988	0.001	992.9	20.7	0.985	0.001	1081.7	11.4	0.995	0.001
1025.1	21.7	0.991	0.001	1064.3	18.6	0.988	0.000	1123.9	10.9	0.995	0.001
1144.6	50.5	0.991	0.001	1164.2	37.1	0.990	0.001	1180.5	24.1	0.996	0.001

Tpx-WT + para-CFT

ew / µm	Δew / µm	$r_{D,LILBID}$	$\Delta r_{D,LILBID}$	ew / µm	Δew / µm	$r_{D,LILBID}$	$\Delta r_{D,LILBID}$	ew / µm	Δew / µm	$r_{D,LILBID}$	$\Delta r_{D,LILBID}$
818.1	37.0	0.367	0.052	823.8	39.1	0.719	0.013	819.1	36.7	0.711	0.029
958.8	34.6	0.592	0.066	956.7	34.8	0.770	0.019	963.5	41.2	0.777	0.033
1069.1	30.0	0.724	0.043	1067.0	26.0	0.833	0.012	1078.2	27.2	0.810	0.005
1158.6	18.1	0.807	0.010	1150.4	19.0	0.862	0.016	1162.3	19.6	0.842	0.017
1226.6	13.9	0.847	0.007	1222.2	18.5	0.899	0.009	1221.3	17.0	0.868	0.005

Tpx-WT + meta-CFT

ew / µm	Δew / µm	$r_{D,LILBID}$	$\Delta r_{D,LILBID}$	ew / µm	Δew / µm	$r_{D,LILBID}$	$\Delta r_{D,LILBID}$	ew / µm	Δew / µm	$r_{D,LILBID}$	$\Delta r_{D,LILBID}$
826.2	41.9	0.753	0.029	811.1	33.1	0.666	0.053	868.1	56.2	0.555	0.049
964.5	38.8	0.860	0.005	935.6	28.0	0.711	0.032	1016.6	27.9	0.694	0.013
1075.3	25.1	0.848	0.010	1062.3	31.4	0.829	0.013	1108.5	19.5	0.738	0.012
1155.8	19.1	0.873	0.004	1155.6	19.6	0.846	0.017	1178.2	15.7	0.766	0.009
1223.3	16.9	0.883	0.003	1222.7	16.9	0.855	0.027	1231.2	12.4	0.807	0.030

Tpx-WT + ortho-CFT

ew / µm	Δew / µm	$r_{D,LILBID}$	$\Delta r_{D,LILBID}$	ew / µm	Δew / µm	$r_{D,LILBID}$	$\Delta r_{D,LILBID}$	ew / µm	Δew / µm	$r_{D,LILBID}$	$\Delta r_{D,LILBID}$
847.5	44.2	0.700	0.034	822.5	39.6	0.816	0.043	879.9	57.1	0.792	0.020
992.3	35.4	0.825	0.025	956.6	35.3	0.924	0.011	1014.1	28.4	0.883	0.022
1088.5	19.3	0.858	0.003	1068.8	27.7	0.921	0.008	1098.3	17.0	0.931	0.008
1151.6	16.5	0.901	0.007	1147.2	18.2	0.908	0.002	1160.6	17.5	0.939	0.002
1219.9	18.7	0.915	0.002	1218.9	21.1	0.909	0.001	1223.6	18.8	0.929	0.009

Tpx-WT + CPT											
**ew / µm**	**Δew / µm**	**$r_{D,LILBID}$**	**$\Delta r_{D,LILBID}$**	**ew / µm**	**Δew / µm**	**$r_{D,LILBID}$**	**$\Delta r_{D,LILBID}$**	**ew / µm**	**Δew / µm**	**$r_{D,LILBID}$**	**$\Delta r_{D,LILBID}$**
821.2	41.6	0.832	0.029	886.9	52.5	0.787	0.029	815.1	38.0	0.802	0.034
954.3	33.3	0.908	0.019	1011.1	24.2	0.769	0.021	942.7	30.1	0.862	0.004
1065.9	27.3	0.918	0.008	1082.7	15.4	0.819	0.008	1051.0	25.0	0.839	0.008
1151.3	21.2	0.922	0.001	1146.9	19.5	0.860	0.007	1143.7	20.7	0.858	0.013
1221.6	18.0	0.937	0.004	1216.2	17.8	0.895	0.007	1219.6	19.4	0.886	0.011
**Tpx-WT + CtFT**											
**ew / µm**	**Δew / µm**	**$r_{D,LILBID}$**	**$\Delta r_{D,LILBID}$**	**ew / µm**	**Δew / µm**	**$r_{D,LILBID}$**	**$\Delta r_{D,LILBID}$**	**ew / µm**	**Δew / µm**	**$r_{D,LILBID}$**	**$\Delta r_{D,LILBID}$**
851.6	61.3	0.981	0.008	789.5	47.9	0.966	0.001	873.8	77.4	0.983	0.005
1018.2	36.2	0.975	0.001	942.5	34.8	0.977	0.004	1028.6	20.4	0.978	0.001
1116.8	21.3	0.980	0.001	1052.3	27.2	0.987	0.001	1089.4	15.5	0.978	0.001
1177.3	12.0	0.984	0.000	1130.1	19.9	0.991	0.001	1140.8	13.9	0.983	0.002
1218.1	11.0	0.985	0.001	1203.3	17.5	0.993	0.001	1202.6	19.6	0.987	0.001

Table S7: Values for $r_{D,LILBID}$ and the corresponding explosion widths resulting from measurements of different Tpx mutants with and without bound para-CFT.

ew / µm	Δew / µm	$r_{D,LILBID}$	$\Delta r_{D,LILBID}$	ew / µm	Δew / µm	$r_{D,LILBID}$	$\Delta r_{D,LILBID}$	ew / µm	Δew / µm	$r_{D,LILBID}$	$\Delta r_{D,LILBID}$
**Tpx-K102E**											
870.7	65.1	0.973	0.002	813.4	56.0	0.986	0.003	837.6	64.8	0.974	0.014
1008.3	24.2	0.979	0.001	967.3	34.8	0.984	0.003	998.2	28.1	0.967	0.007
1090.8	19.9	0.984	0.002	1063.1	18.1	0.989	0.000	1089.0	21.7	0.971	0.003
1152.8	16.0	0.987	0.000	1133.1	20.9	0.987	0.000	1157.9	16.9	0.973	0.002
1207.7	14.4	0.989	0.001	1205.9	17.5	0.988	0.001	1212.1	13.6	0.974	0.002
**Tpx-K102E + para-CFT**											
798.3	27.2	0.928	0.005	846.0	50.1	0.859	0.047	919.0	63.0	0.912	0.047
904.9	32.3	0.929	0.005	982.9	31.6	0.905	0.006	1045.4	21.2	0.937	0.022
1018.3	30.8	0.943	0.004	1084.5	23.8	0.933	0.006	1114.3	18.1	0.947	0.018
1136.6	27.4	0.947	0.005	1163.5	19.7	0.946	0.004	1177.0	15.2	0.954	0.016
1220.2	18.5	0.962	0.001	1224.8	16.7	0.952	0.001	1228.8	14.3	0.959	0.014
**Tpx-K102A**											
794.4	28.2	0.978	0.001	819.0	36.2	0.993	0.002	935.0	56.2	0.987	0.009
882.8	21.2	0.979	0.001	946.8	34.8	0.992	0.001	1070.8	25.6	0.988	0.001
952.1	18.3	0.982	0.001	1061.9	26.4	0.991	0.002	1146.4	15.8	0.989	0.005
1018.0	19.9	0.986	0.000	1149.4	21.4	0.993	0.000	1197.4	13.1	0.992	0.001
1111.1	35.1	0.990	0.002	1222.9	17.0	0.993	0.000	1237.2	8.4	0.994	0.001
**Tpx-K102A + para-CFT**											
841.3	44.2	0.853	0.031	940.8	73.5	0.928	0.018	882.5	61.0	0.842	0.025
971.7	29.6	0.893	0.013	1084.9	19.9	0.948	0.003	1043.4	32.6	0.874	0.006
1070.6	23.8	0.904	0.006	1137.8	10.9	0.955	0.003	1133.0	18.2	0.890	0.011
1148.2	17.2	0.912	0.004	1173.6	10.7	0.957	0.003	1188.3	12.5	0.904	0.003
1216.0	20.0	0.924	0.005	1222.5	15.8	0.961	0.002	1230.8	13.0	0.919	0.005

Tpx-W39A											
ew / µm	Δew / µm	$r_{D,LILBID}$	$\Delta r_{D,LILBID}$	ew / µm	Δew / µm	$r_{D,LILBID}$	$\Delta r_{D,LILBID}$	ew / µm	Δew / µm	$r_{D,LILBID}$	$\Delta r_{D,LILBID}$
971.0	72.9	0.978	0.004	780.8	20.2	0.988	0.001	827.1	46.6	0.991	0.006
1106.3	21.0	0.990	0.005	855.1	21.2	0.989	0.005	976.4	35.3	0.991	0.004
1165.6	11.6	0.987	0.006	937.4	18.8	0.992	0.004	1083.7	23.9	0.991	0.004
1205.0	10.3	0.989	0.001	994.7	15.2	0.991	0.003	1159.1	18.1	0.997	0.002
1238.7	8.8	0.990	0.002	1077.8	38.6	0.992	0.002	1226.0	17.5	0.995	0.002

Tpx-W39A + para-CFT											
ew / µm	Δew / µm	$r_{D,LILBID}$	$\Delta r_{D,LILBID}$	ew / µm	Δew / µm	$r_{D,LILBID}$	$\Delta r_{D,LILBID}$	ew / µm	Δew / µm	$r_{D,LILBID}$	$\Delta r_{D,LILBID}$
809.4	37.1	0.967	0.002	847.9	51.7	0.983	0.014	865.7	56.6	0.975	0.004
930.0	27.4	0.969	0.002	997.0	30.8	0.984	0.003	1015.9	28.7	0.976	0.007
1028.3	29.7	0.971	0.003	1085.3	19.1	0.983	0.002	1097.3	18.5	0.986	0.002
1125.2	22.1	0.973	0.001	1154.2	20.3	0.986	0.001	1157.5	14.2	0.979	0.004
1208.1	21.4	0.972	0.002	1225.2	15.4	0.986	0.001	1216.6	19.0	0.985	0.001

Table S8: Values for $r_{D,LILBID}$ and the corresponding explosion widths resulting from measurements of Roquin and LBE.

**Roquin + LBE**							
**ew / µm**	**Δew / µm**	$r_{D,LILBID}$	$\Delta r_{D,LILBID}$	**ew / µm**	**Δew / µm**	$r_{D,LILBID}$	$\Delta r_{D,LILBID}$
765.8	5.1	0.629	0.051	1015.1	3.1	0.697	0.031
775.1	4.7	0.644	0.047	1025.5	6.3	0.677	0.063
785.7	5.8	0.649	0.058	1035.8	3.6	0.712	0.036
792.6	2.2	0.670	0.022	1043.7	3.4	0.693	0.034
804.4	4.0	0.665	0.040	1054.5	2.6	0.695	0.026
815.1	4.5	0.629	0.045	1064.3	5.0	0.702	0.050
824.6	4.8	0.651	0.048	1075.2	6.2	0.699	0.062
834.8	4.9	0.658	0.049	1086.0	3.1	0.728	0.031
844.2	5.0	0.656	0.050	1094.6	3.7	0.734	0.037
855.4	4.7	0.666	0.047	1105.6	7.7	0.701	0.077
867.5	4.1	0.663	0.041	1116.3	4.6	0.706	0.046
874.8	3.3	0.665	0.033	1124.5	2.2	0.726	0.022
885.0	3.1	0.669	0.031	1135.8	6.0	0.698	0.060
897.0	2.9	0.652	0.029	1144.3	4.4	0.714	0.044
905.6	2.5	0.671	0.025	1154.5	3.2	0.720	0.032
915.6	3.6	0.671	0.036	1164.3	4.3	0.711	0.043
923.3	5.0	0.644	0.050	1175.4	2.6	0.729	0.026
933.5	3.9	0.687	0.039	1184.9	6.7	0.700	0.067
945.0	3.3	0.686	0.033	1194.4	3.4	0.713	0.034
954.8	3.2	0.674	0.032	1204.1	7.1	0.705	0.071
964.9	3.4	0.677	0.034	1213.4	4.2	0.697	0.042
974.1	3.7	0.683	0.037	1225.4	5.3	0.718	0.053
985.5	4.1	0.670	0.041	1233.8	4.6	0.715	0.046
995.6	3.0	0.691	0.030	1245.1	4.7	0.718	0.047
1005.6	2.9	0.690	0.029				

Table S9: Values for $r_{D,LILBID}$ and the corresponding explosion widths resulting from measurements of BSA with the charge state correction determined either *via* dilution or crosslinking.

**BSA dilution approach**				**BSA crosslinking approach**			
**ew / µm**	**Δew / µm**	$r_{D,LILBID}$	$\Delta r_{D,LILBID}$	**ew / µm**	**Δew / µm**	$r_{D,LILBID}$	$\Delta r_{D,LILBID}$
860.3	74.9	0.809	0.031	860.3	74.9	0.804	0.009
1044.5	32.9	0.824	0.010	1044.5	32.9	0.812	0.001
1128.8	12.6	0.851	0.017	1128.8	12.6	0.831	0.007
1176.7	13.9	0.876	0.005	1176.7	13.9	0.838	0.005
1219.8	11.1	0.879	0.009	1219.8	11.1	0.847	0.008

Supplementary Figures

strA	5' ATTGTAGTTTTTTTGAGTTGATTATGTTTTTAGTA 3'	K_D / nM [1]
cstrA(16-26)	3' TCAACTAATAC 5'	370 ± 50
cstrA(18-26)	3' AACTAATAC 5'	8500 ± 700
cstrA(21-35)	3' TAATACAAAAATCAT 5'	31.2 ± 0.8
cstrA(26-35)	3' CAAAAATCAT 5'	3900 ± 500

strB	5' TTTTGTGAGATTACGGAACCTTTTTTTTTTTTTGT 3'	K_D / nM [1]
cstrB(5-16)	3' CACTCTAATGCC 5'	20.1 ± 1.8
cstrB(6-15)	3' ACTCTAATGC 5'	353 ± 7
cstrB(8-16)	3' TCTAATGCC 5'	2120 ± 60

strC	5' TTTTATCGGCTCTTCTCATTTTATTTTATTTTGTT 3'	K_D / nM [1]
cstrC(7-16)	3' GCCGAGAAGA 5'	29 ± 7
cstrC(8-15)	3' CCGAGAAG 5'	418 ± 16

Figure S1: Sequences and K_Ds of the DNAs used for qLILBID calibration.
Sequences of three single-stranded DNAs (ssDNA), strA, strB, and strC, each consisting of 35 nucleotides (nt), displayed in the 5' to 3' orientation. Below each large strand, shorter ssDNA sequences (cstrA, cstrB, cstrC) are aligned in the 3' to 5' orientation to highlight the respective complementary binding regions to the larger strands within a distinct segment indicated by nucleotide position in parentheses: cstrA(16-26), cstrA(18-26), cstrA(21-35), and cstrA(26-35) bind to strA; cstrB(5-16), cstrB(6-15), and cstrB(8-16) bindto strB; and cstrC(7-16) and cstrC(8-15) bind to strC. Dissociation constant (K_D) values are listed on the right. All K_D values are taken from Young et al.[1]

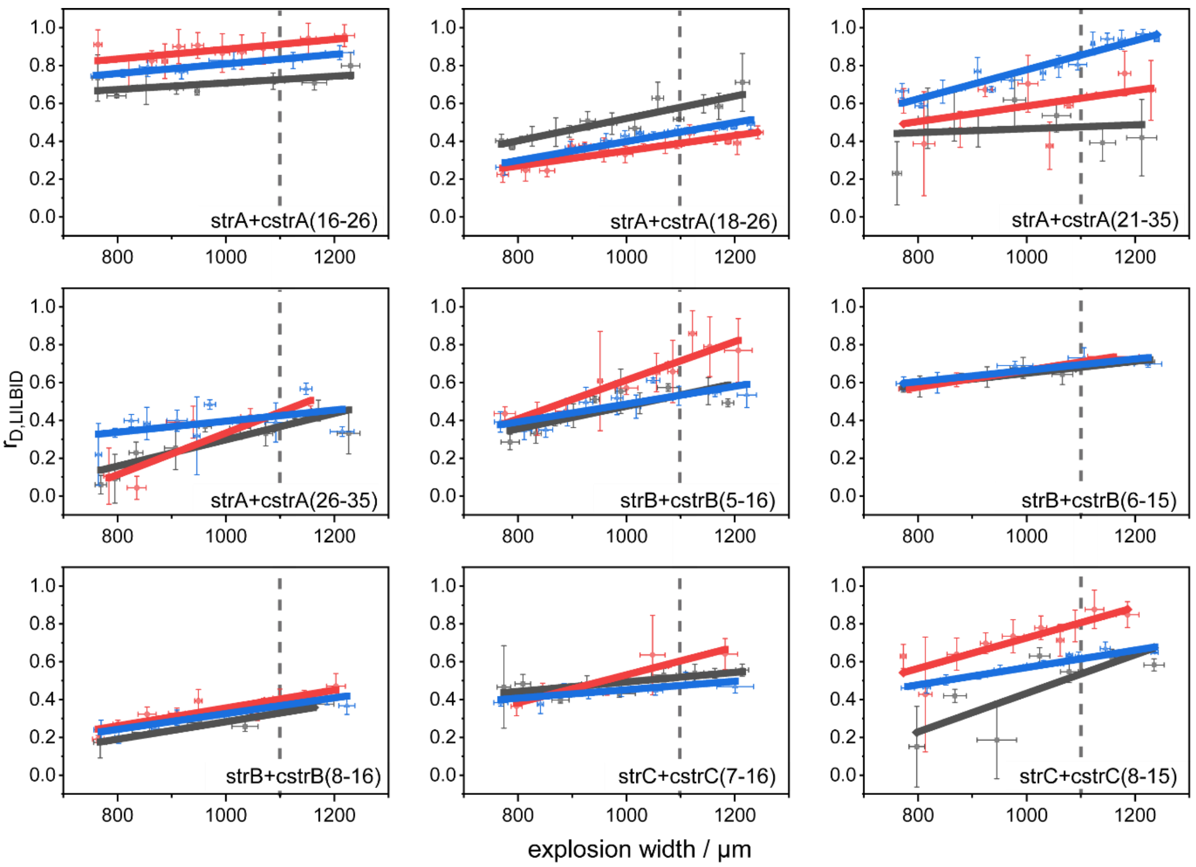

Figure S2: Dissociation plots of dsDNAs used for qLILBID affinity calibration.
Dissociation plots of strA, strB and strC and shorter strands complementary to different parts of the main strand (see **Figure S1**). The relative amount of monomer is plotted against the explosion width (plume size) of the droplet explosion and fitted linearly. Plotted data points are given in **Supplementary Table 4.** Resulting fit parameters and percentage of dissociated complex at 1100 µm explosion width are given in **Supplementary Table 1**. For all samples, the measurements were repeated three times (shown in black, blue and red).

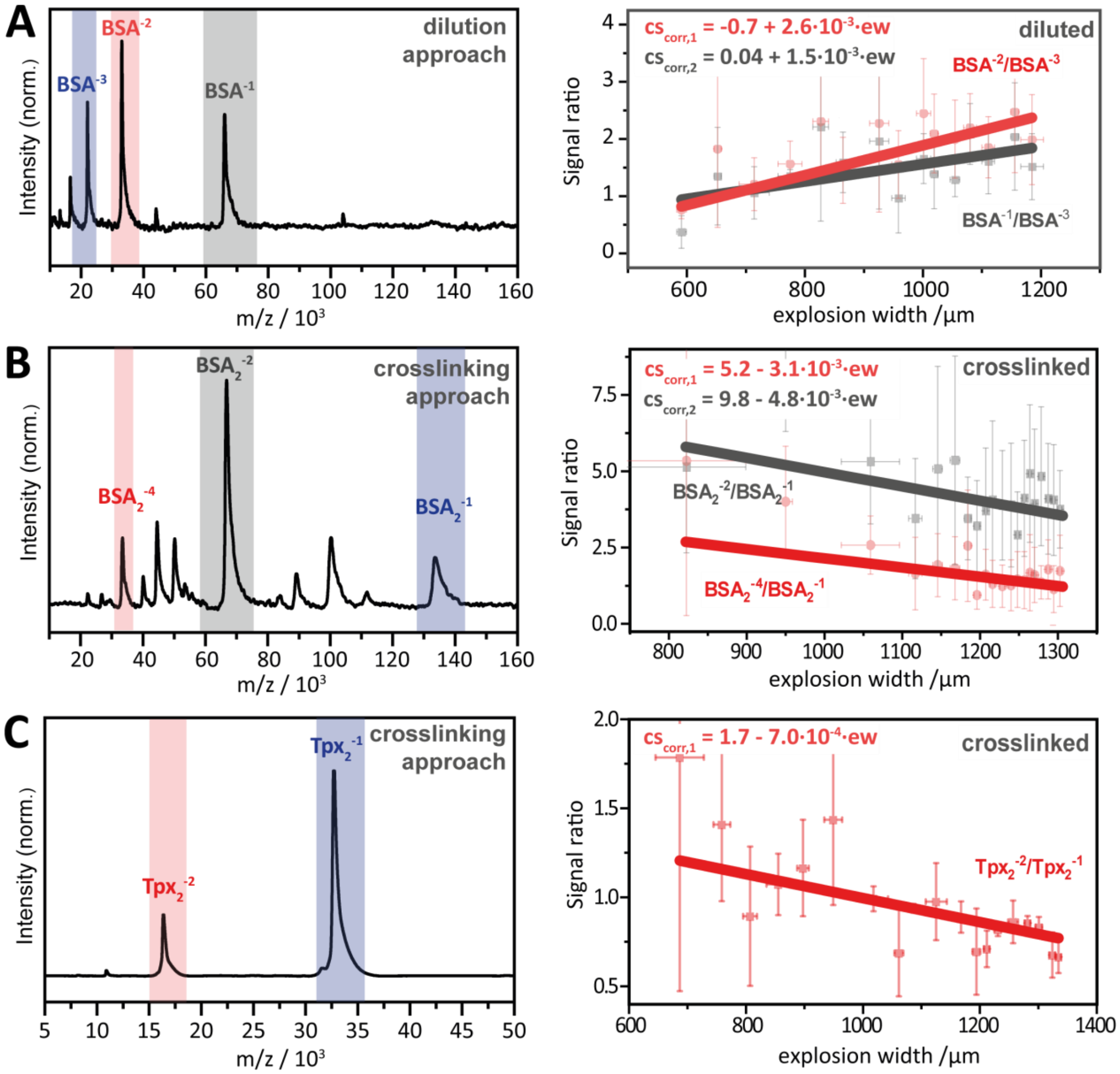

Figure S3: Dilution and crosslinking approach with different protein homooligomers.
Left: Spectra of either the crosslinked or diluted protein sample (Bovine Serum Albumin, BSA; Tryparedoxin, Tpx), where only one of the relevant species (either monomer or dimer) is visible. The relevant peak areas are highlighted. Blue is the reference peak, where no peak overlap in an equilibrium is expected. *Right:* The signal ratios gained from diluted or crosslinked spectra plotted against the explosion width. This is used to determine the charge state ratios as detailed in the main text. The fit yields a charge state correlation function (cs_{corr}), which is dependent on the explosion width (ew). Plotted data points are given in **Supplementary Table 5. (A)** *Left:* Spectrum of diluted BSA (100 nM) with only monomeric species. *Right*: The signal ratio of doubly charged BSA (red) and singly charged BSA (grey) compared to the three times charged BSA (blue in spectrum) are plotted against the explosion width. **(B)** *Left:* Spectrum of the crosslinked BSA shows no monomeric species. The highlighted areas correspond to the relevant peak m/z. *Right*: The signal ratio of the four times charged BSA dimer (red) and two times charges dimer (grey) to the singly charged BSA dimer (blue in spectrum) are plotted against the explosion width. **(C)** *Left:* Spectrum of the crosslinked Tpx showing no monomeric species. The highlighted areas correspond to the relevant peak m/z. *Right*: Signal ratio gained from the diluted spectra to determine the monomeric and dimeric share in overlapping peaks. The signal ratio of the two times charged Tpx dimer (red) to the singly charged Tpx dimer (blue in spectrum) is plotted against the explosion width.

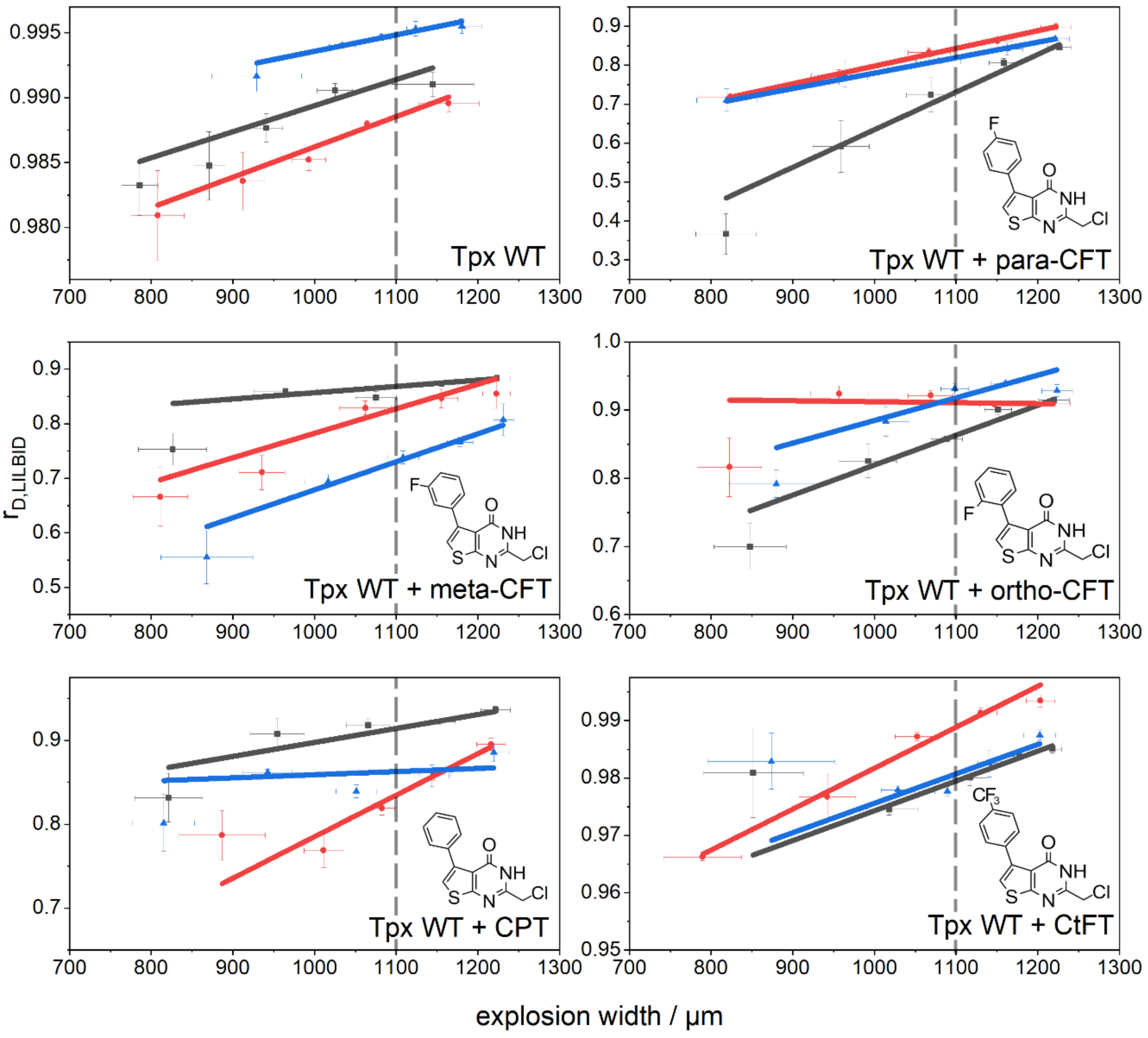

Figure S4: Dissociation plots of Tpx WT with and without different covalently bound molecular glues. Depending on the molecular glue, each induced Tpx dimer has a different affinity.[2] The relative amount of monomer is plotted against the explosion width (plume size of the droplet explosion) and fitted linearly. Plotted data points are given in **Supplementary Table 6.** Resulting fit parameters and percentage of dissociated complex at 1100 µm explosion width are given in **Supplementary Table 2**. For all samples, the measurements were repeated three times (each replicate shown in black, blue and red, respectively).

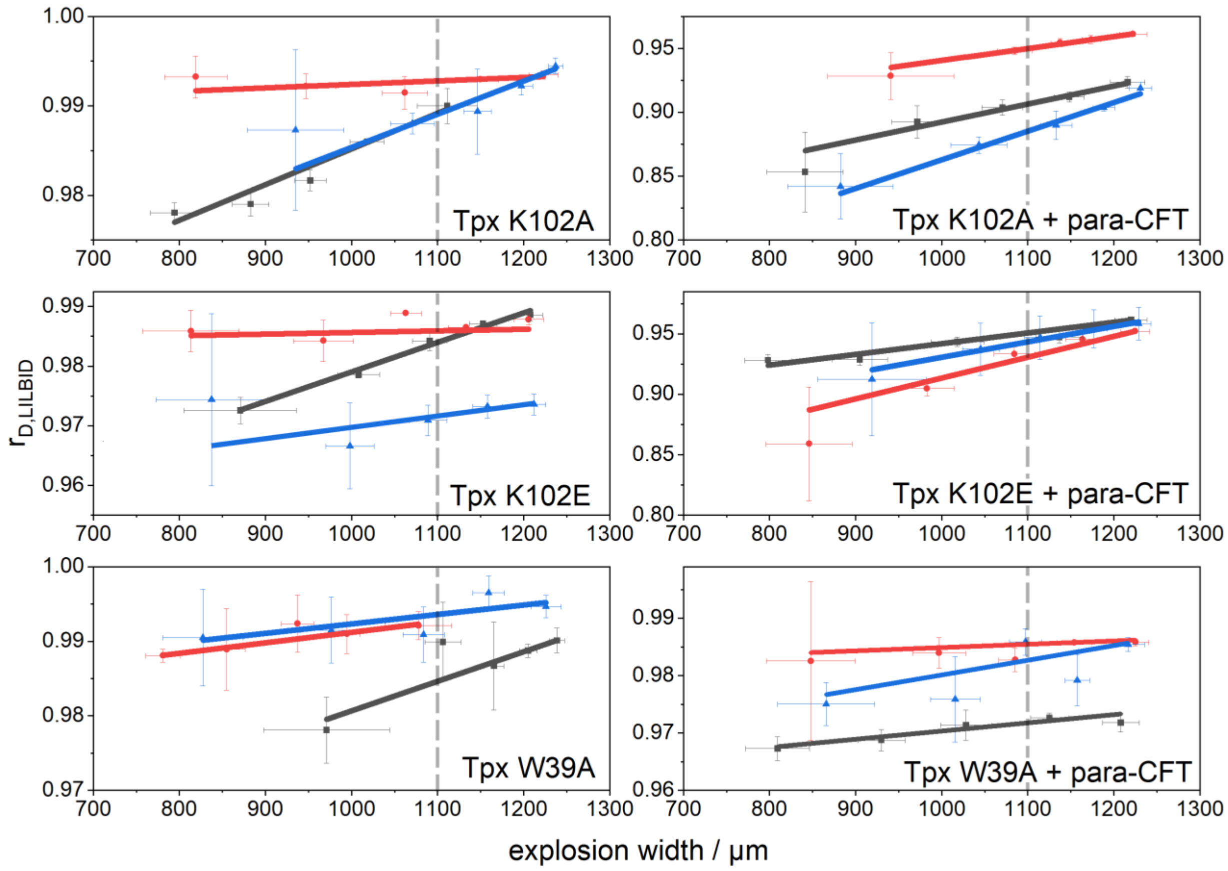

Figure S5: Dissociation plots of Tpx K102 and W39 mutants with and without the covalently bound para-CFT inhibitor. The mutants display significantly reduced dimer affinity compared to the WT. The relative amount of monomer is plotted against the explosion width (plume size of the droplet explosion) and fitted linearly. Plotted data points are given in **Supplementary Table 7.** Resulting fit parameters and percentage of dissociated complex at 1100 µm explosion width are given in **Supplementary Tables 2 and 3**. For all samples, the measurements were repeated three times (each replicate shown in black, blue and red, respectively).

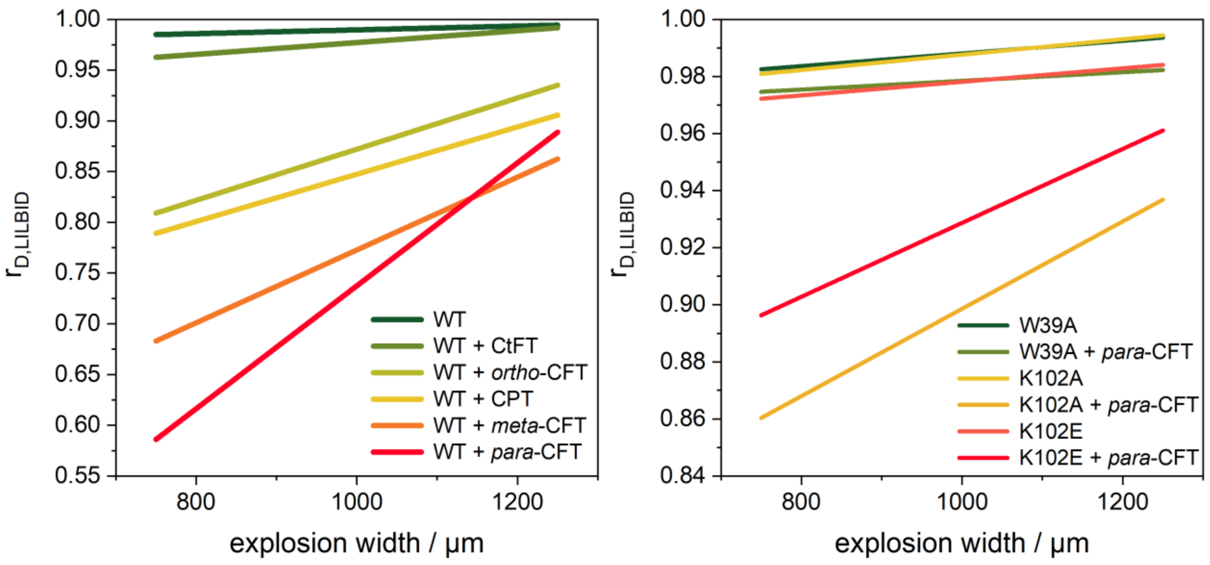

Figure S6: Mean dissociation plots of Tpx WT and mutants with and without different covalently bound molecular glues

Dissociation plots of Tpx WT and mutants alone and in complex with different covalent dimerizers, (para-CFT for all mutants, para-CFT, meta-CFT, ortho-CFT, CtFT and CPT for the WT). Depending on the molecular glue and the mutation, each induced Tpx dimer has a different affinity.[2] The resulting mean relative amounts of monomer of the three linear fits (**Supplementary Figures S4 and S5**) of each species is plotted against the explosion width (plume size of the droplet explosion) and fitted linearly. Graphs with a high $r_{D,LILBID}$ represent species with low binding affinity and those with low $r_{D,LILBID}$ correspond to species with high binding affinity.

Calculation of $r_{D,LILBID}$ from peak integrals

In BSA and Tpx spectra, we see charge state overlap. For BSA, the peaks at ca. 66 kDa and 33 kDa are peaks of overlapping species ($Dimer^{2-}$/$Monomer^{1-}$ and $Dimer^{4-}$/$Monomer^{2-}$ respectively) while for Tpx the peak at ca. 16 kDa is a peak of overlapping species ($Dimer^{2-}$/$Monomer^{1-}$). To distinguish between monomeric and dimeric contributions within the overlapping peaks, we applied charge state correlation functions to resolve their respective distributions. (cs_{corr}, **Figure S3**).

Crosslinking approach: (**Figure S3B, S3C**)

$$\int Dimer^{2-} = cs_{corr,1}(ew) \cdot \int Dimer^{1-} \tag{S1}$$

$$\int Monomer^{1-} = \int (Monomer^{1-} + Dimer^{2-}) - cs_{corr,1}(ew) \cdot \int Dimer^{1-} \tag{S2}$$

$$\int Dimer^{4-} = cs_{corr,2}(ew) \cdot \int Dimer^{1-} \tag{S3}$$

$$\int Monomer^{2-} = \int (Monomer^{2-} + Dimer^{4-}) - cs_{corr,2}(ew) \cdot \int Dimer^{1-} \tag{S4}$$

Dilution approach: (**Figure S3A**)

$$\int Monomer^{1-} = cs_{corr,1}(ew) \cdot \int Monomer^{3-} \tag{S5}$$

$$\int Dimer^{2-} = \int (Monomer^{1-} + Dimer^{2-}) - cs_{corr,1}(ew) \cdot \int Monomer^{3-} \tag{S6}$$

$$\int Monomer^{2-} = cs_{corr,2}(ew) \cdot \int Monomer^{3-} \tag{S7}$$

$$\int Dimer^{4-} = \int (Monomer^{2-} + Dimer^{4-}) - cs_{corr,2}(ew) \cdot \int Monomer^{3-} \tag{S8}$$

For all approaches and measurements:

$$r_{D,LILBID} = \frac{\sum_{cs} \int Monomer^{cs}}{\sum_{cs} \int Monomer^{cs} + \sum_{cs} \int Dimer^{cs}} \tag{S9}$$

Derivation of the K_D from $r_{D,solution}$ for heterodimers (equation 4a):

$$r_{D,solution} = \frac{n(M_1)}{n(M_1 + D)} = \frac{n(M_2)}{n(M_2 + D)} \quad (3)$$

Using the same concentration for both binding partners ($c(M_1) = c(M_2) = c(M)$ and c_{total} as the total concentration of each protein: $c_{total} = c(M) + c(D)$

$$r_{D,solution} = \frac{c(M)}{c_{total}} \quad \text{(S10a)}$$

$$\Rightarrow c(M) = r_{D,solution} \cdot c_{total} \quad \text{(S10b)}$$

Insert into the definition for K_D:

$$K_D = \frac{c(M_1) \cdot c(M_2)}{c(D)} = \frac{c(M)^2}{c(D)} = \frac{c(M)^2}{c_{total} - c(M)} \quad \text{(S11)}$$

$$K_D = \frac{\left(r_{D,solution} \cdot c_{total}\right)^2}{c_{total} - r_{D,solution} \cdot c_{total}} = \frac{c_{total} \cdot {r_{D,solution}}^2}{1 - r_{D,solution}} \quad \text{(S12)}$$

Rearranging the equation enables calculation of expected $r_{D,solution}$ values for samples with known literature K_D values for calibration (see Table S1):

$$r_{D,solution} = -\frac{K_D}{2\,c_{total}} + \sqrt{\left(\frac{K_D}{2\,c_{total}}\right)^2 + \frac{K_D}{c_{total}}} \quad \text{(S12b)}$$

Derivation of the K_D from $r_{D,solution}$ for homodimers (equation 4b):

$$r_{D,solution} = \frac{n(M)}{n(M + D)} = \frac{c(M)}{c(M) + c(D)} \quad \text{(S13)}$$

With $c(D) = \frac{1}{2}\left(c_{total} - c(M)\right)$:

$$r_{D,solution} = \frac{c(M)}{c(M) + \frac{1}{2}\left(c_{total} - c(M)\right)} = \frac{2 \cdot c(M)}{c(M) + c_{total}} \quad \text{(S14a)}$$

$$c(M) = \frac{r_{D,solution} \cdot c_{total}}{2 - r_{D,solution}} \quad \text{(S14b)}$$

$$c(D) = \frac{1}{2}\left(c_{total} - \frac{r_{D,solution} \cdot c_{total}}{2 - r_{D,solution}}\right) = \frac{1}{2} c_{total} \cdot \left(1 - \frac{r_{D,solution}}{2 - r_{D,solution}}\right) \quad \text{(S15a)}$$

$$c(D) = \frac{1}{2} c_{total} \cdot \left(\frac{2 - r_{D,solution} - r_{D,solution}}{2 - r_{D,solution}}\right) = c_{total} \cdot \left(\frac{1 - r_{D,solution}}{2 - r_{D,solution}}\right) \quad \text{(S15b)}$$

Insert into the definition for K_D:

$$K_D = \frac{c(M) \cdot c(M)}{c(D)} = \frac{c(M)^2}{c(D)} = \frac{\left(\frac{r_{D,solution} \cdot c_{total}}{2 - r_{D,solution}}\right)^2}{c_{total} \cdot \left(\frac{1 - r_{D,solution}}{2 - r_{D,solution}}\right)} \tag{S16a}$$

$$K_D = \frac{c_0 \cdot \left(\frac{r_{D,solution}}{2 - r_{D,solution}}\right)^2}{\left(\frac{1 - r_{D,solution}}{2 - r_{D,solution}}\right)} = \frac{\frac{c_{total}}{2 - r_{D,solution}} \cdot r_{D,solution}{}^2}{1 - r_{D,solution}} \tag{S16b}$$

$$K_D = \frac{c_{total} \cdot r_{D,solution}{}^2}{r_{D,solution}^2 - 3 \cdot r_{D,solution} + 2} \tag{S16c}$$

Materials and Methods

DNA preparation. ssDNA sequences for calibration originate from Young et al.[1] and were purchased from Thermo Fisher Scientific as desalted, dry custom oligonucleotides (see Table 1 for sequences and K_Ds). The set of ssDNAs comprised three non-self-complementary strands with lengths of 35 nucleobases, as well as nine shorter ssDNAs complementary to specific parts of the larger strands and differing in their length and C-G content. Each ssDNA was dissolved in a buffer containing 0.5 mM $MgHPO_4$ at pH 7.2 to reach a final concentration of 10 µM. To create dsDNAs with nM to low-µM affinities, equimolar amounts of long and short, complementary ssDNAs were annealed at 95°C for 10 minutes and gradually cooled to room temperature over a period of several hours.

BSA preparation. BSA (bovine serum albumin) was purchased from Sigma-Aldrich as a lyophilized powder and dissolved in 100 mM NH_4CH_3COO. To get solely dimeric BSA as reference, 20 µl of 30 µM BSA was crosslinked by incubating it with 2 mM 1-ethyl-3-[3-dimethylaminopropyl]-carbodiimid-hydrochlorid (EDC) in Tris-HCl buffer at pH 7 overnight and separating the dimer from unreacted BSA monomer and EDC by repeated dilution and filtering over an Amicon Ultra Centrifugal Filter unit (Sigma-Aldrich) with a cutoff of 100 kDa. Shortly before qLILBID-MS measurements, the crosslinked as well as the untreated BSA were desalted with Zeba™ Micro Spin desalting columns (Thermo Fisher Scientific) equilibrated with 200 mM NH_4CH_3COO at pH 7.5.

Tpx purification and derivatization. *Trypanosoma brucei* Tryparedoxin (Tpx) was heterologously overexpressed as a proteolytically cleavable, His_6-tagged Trx-fusion protein in *E. coli* BL21GoldDE3 cells (Agilent) and purified via Ni-affinity (Qiagen) and size exclusion chromatography (SEC) (HiLoad16/600 Superdex75 pg column, Cytiva) as described previously.[2] For covalent modification, 100 µM Tpx were incubated with 4 mM tris(2-carboxyethyl)-phosphine (TCEP) and a 2-fold excess of dimerizer (4 mM in DMSO) or a 4-fold excess of crosslinker 1,8-bismaleimidodiethyleneglycol ($BM(PEG)_2$, 10 mM in DMSO) at 25 °C for 30 min, and purified via SEC (Superdex75 Increase 10/300 GL column (Cytiva) at 4 °C. All proteins were stored in Tpx buffer (25 mM NaPi, 150 mM NaCl, pH 7.5) at -20°C.

ITC measurements. Dissociation constants (K_Ds) of Tpx constructs were determined via dilution ITC using MicroCal PEAQ-ITC instruments (Malvern Panalytical).[2] Tpx constructs in Tpx buffer were injected into sample cells with pure buffer, and the measured heat signals were analyzed using the MicroCal PEAQ-ITC Analysis software (Malvern Panalytical). Each titration was performed in a technical triplicate at room temperature.[2]

LILBID measurements. LILBID-MS spectra of dsDNA and protein homodimers were acquired at room temperature using a custom-built mass spectrometer equipped with a LILBID ion source and Time-of-Flight (ToF) detector described in Morgner et al.[3] In brief, 5 µl of the aqueous sample are injected into a droplet generator and droplets with concentrations between 0.1 to 30 µM are emitted through a glass capillary with a nozzle width of 50 µm. These droplets are then transferred into a vacuum environment of approximately 10^{-5} mbar and exposed to a 2.8 µm laser pulse (Innolas Spitlight 400, Continuum PowerLite 8000) lasting about 6 ns and with a pulse energy between 7 and 20 mJ. This process causes a rapid expansion of the droplets, enabling ions from the sample to transition into the gas phase and be analyzed based on their mass-to-charge ratio (m/z). The resulting droplet plume is illuminated by a Minilite I laser (Continuum, San Jose, USA) 5 µs after the infrared (IR) laser pulse and captured by a DFK 23UP031 camera (Imaging Source, Bremen, Germany). Here, each droplet yielded a mass spectrum and an image of the corresponding plume. For all samples 500 to 2000 spectra and plume images were collected and then analyzed. The exact amount of spectra recorded were dependent on the quality of the measurements which depends on factors such as S/N and stability of the droplet.

LILBID Data Analysis. Analysis of LILBID spectra and plume images was performed using a custom Python application developed for qLILBID experiments. Videos were first processed to extract the explosion widths of the desorbed droplets. For each video, a background image was generated by computing the 18th percentile across all frames, which was subtracted from all subsequent frames to suppress static features. After grayscale conversion and binary thresholding, the largest contour in each frame was identified, and the two furthest-apart contour points were determined by pairwise Euclidean distance. Their separation was taken as the explosion width (in pixels) and was recorded for every frame together with the plume orientation angle.

Mass spectra were imported from .dat files, from which the m/z axis and individual-shot intensity traces were extracted within a sample depenent m/z interval. Spectra and explosion-width files were matched through numeric suffixes in the filenames. For analysis, all spectra were sorted according to their corresponding explosion widths and then summed batch-wise to enhance the signal-to-noise ratio. For each batch, the summed spectrum was normalized to its intensity range, and the representative explosion width was calculated as the mean of all frames included in that batch, corrected by the pixel size (10.4 µm/pixel).

Peak areas were quantified using sample dependent m/z start and end values with an allowed variation. For each peak, the program identified the nearest indices in the m/z axis and determined local minima around the boundaries to define the integration window. Baseline noise was estimated from flanking regions, and peak areas were integrated after baseline subtraction. For every batch, the program exported a CSV file containing the batch-mean explosion width and the integrated peak areas of all defined spectral features.

References

1. Young, P., Hense, G., Immer, C., Wöhnert, J. & Morgner, N. LILBID laser dissociation curves: a mass spectrometry-based method for the quantitative assessment of dsDNA binding affinities. *Sci. Rep.* **10**, 20398 (2020).

2. Schwegler, E. *et al.* Inhibitor fluorination pattern tunes chemically induced protein dimerization. *bioRxiv* (2025) doi:10.1101/2025.07.23.666362.

3. Morgner, N., Barth, A. H. & A, B. B. A New Way To Detect Noncovalently Bonded Complexes of Biomolecules from Liquid Micro-Droplets by Laser. 109–114 (2006).
